# A Fast and Generalizable Deep Neural Network for the Detection of Atrial Fibrillation

**DOI:** 10.3390/bioengineering13101174

**Published:** 2026-10-08

**Authors:** Harshit Mishra, Farhan Adam, Nachiket Makwana, Pradyot Tiwari, Subramani Kandasamy, K. V. S. Hari

**Affiliations:** 1 Gauze, Fynd, Mumbai 400093, India; 2 Department of Bioengineering, Indian Institute of Science, Bengaluru 560012, India; 3 Department of Cardiology, Waikato District Health Board, Hamilton 3204, New Zealand; 4 Surgical Critical Care Unit, Department of Critical Care Medicine, Christian Medical College, Vellore 632004, India; 5 Department of Electrical Communication Engineering, Indian Institute of Science, Bengaluru 560012, India

**Keywords:** atrial fibrillation, deep learning, ECG analysis, convolutional neural networks, medical signal processing, cardiovascular disease detection

## Abstract

Atrial fibrillation (AFib) is the most prevalent sustained cardiac arrhythmia, yet most deep learning detectors are validated on a single cohort and few report uncertainty. We developed a compact 14M-parameter convolutional neural network for binary AFib classification from 10 s, 12-lead ECG at 500 Hz, trained exclusively on the HDXML dataset (67,432 ECGs; 9628 AFib) with on-the-fly augmentation for noise, drift and missing channels. Using one unchanged checkpoint, we evaluated six independently sourced public cohorts totaling over 1.25 million recordings, including MIMIC-IV critical care and two ambulatory Holter cohorts. All metrics are reported with 95% confidence intervals from a cluster bootstrap at the patient, recording or subject levels. ROC–AUC exceeded 0.95 in every cohort, from 0.960 (95% CI 0.956–0.963) on CODE-15 to 0.999 (0.998–0.999) on SPH, including 0.966 (0.965–0.967) on MIMIC-IV with 75.3% sensitivity and 97.7% specificity, 0.963 (0.929–0.987) on CPSC 2021 and 0.995 (0.988–0.999) on MIT-BIH. Performance was stable under progressive lead masking without retraining, and median inference took 110 ms per segment on a server-class CPU without a GPU. A compact network trained on one curated source can therefore generalize across hospitals, countries and recording hardware. This study is retrospective; prospective validation is required before clinical use.

## 1. Introduction

Atrial fibrillation (AFib) is the most common sustained cardiac arrhythmia, affecting over 33.5 million persons worldwide and projected to reach 17.9 million in Europe and 12.1 million in the USA by 2030 [1,2]. It accounts for nearly 454,000 US hospitalizations and 158,000 deaths annually and adds up to $26 billion in direct health-care spending [3,4]. The problem is magnified in critical care, where new-onset AFib occurs in 5–15% of intensive-care admissions and independently raises 30-day mortality by 40–60% [5,6]; AFib also confers a five-fold stroke risk, triples heart-failure incidence and doubles all-cause mortality [7]. Guidelines therefore emphasize early rhythm identification to guide anticoagulation and rhythm control [8,9], yet manual ECG interpretation is resource-intensive and error-prone, motivating automated detectors deployable at the bedside or on wearables.

Wearable screening has been evaluated at scale: the Apple Heart Study enrolled 419,297 participants and the Fitbit Heart Study 455,699, both establishing that consumer devices can surface previously undiagnosed atrial fibrillation [10,11]. The evidence remains qualified—the US Preventive Services Task Force found it insufficient to recommend screening in asymptomatic adults [12], and device accuracy has been shown to drift over time [13]—so a detector intended for this setting must be judged on specificity and predictive value at the prevalence of the screened population, not on discrimination alone.

**Evolution of automated AFib detection.** First-generation algorithms exploited heart rate variability: Tateno and Glass [14] achieved 94.4% sensitivity and 97.2% specificity from RR-interval histograms, and Dash et al. surpassed 98% accuracy on their internal test sets by combining time- and frequency-domain descriptors [15]. Later, entropy measures [16] improved robustness, but all such approaches depended on expert-driven feature engineering and were prone to failure under unseen conditions.
**Deep learning era.** Rajpurkar et al. framed arrhythmia detection as a sequence-to-sequence task over raw single-lead ECG using a 34-layer 1-D ResNet [17], subsequently validated at cardiologist-level F1 across twelve rhythm classes in 53,549 ambulatory patients [18], and Attia et al.’s “AI-ECG” adapted the same convolutional philosophy to 12 leads with progressively wider receptive fields [19]. Ribeiro et al. ported the 2-D residual network to one-dimensional 12-lead input for multi-label diagnosis of six abnormalities [20]. Moving beyond pure CNNs, Natarajan et al.’s “wide-and-deep Transformer” combines a convolutional front-end and an eight-layer Transformer encoder (d=256) with 22 handcrafted features before the final classifier [21]. Most such studies are nevertheless evaluated at a single site, leaving cross-cohort generalization and deployability open.
**Our contribution.** We present a fast 14M-parameter CNN trained on a single carefully curated corpus of 67,432 12-lead ECGs—the Heart Disease HDXML Strain dataset of Tse et al. [22]—and externally validated across six public datasets, including MIMIC-IV critical-care recordings. Channel attention and dual-path fusion permit identical weights to process anything from a single-lead wearable trace to a full 12-lead ECG, serving both outpatient monitors and ICU telemetry.

## 2. Materials and Methods

### 2.1. Dataset Preparation

We used nine open-access ECG datasets. Training used the HDXML corpus [22] exclusively; external validation used six independently sourced cohorts—MIMIC-IV ECG [23], CODE-15 [24], CPSC 2021 [25], PhysioNet/CinC 2021 [26], SPH [27] and the MIT-BIH Arrhythmia Database [28]—together comprising over 1.25 million recordings (Table 1). Chapman–Shaoxing [29] and the PhysioNet/CinC 2020 challenge set (Offline-TS) [30] are reported separately in Table 2, their records being contained within PhysioNet/CinC 2021, and MedalCare-XL [31] contributed synthetic samples used only in the supplementary multi-dataset experiment. “External validation” here means retrospective testing on datasets excluded from training, not prospective clinical validation.

**Cohort independence.** Relationships between cohorts were established from release provenance and identifier ranges, not from record names, which differ between releases of the same underlying data. PhysioNet/CinC 2021 is assembled from eight source databases and contains both the Chapman–Shaoxing collection (identifiers JS00001–JS10646) and the six databases constituting the PhysioNet/CinC 2020 challenge set; both are therefore subsets of it and are not counted as independent cohorts. The six cohorts above draw on separate institutions, countries and acquisition programs. CPSC 2021 shares a challenge consortium with the CPSC 2018 database inside PhysioNet/CinC 2021 but comprises separately collected Holter recordings at a different sampling rate and lead count; no shared identifiers exist, so overlap cannot be formally excluded. Its 1436 recordings originate from 105 patients, whose identifiers are encoded in the record names; 1435 are evaluated here, one record being shorter than a single 10 s window.
**Ambulatory cohorts.** CPSC 2021 and MIT-BIH are two-lead Holter recordings, evaluated in non-overlapping 10 s windows. Available leads were placed at their standard 12-lead positions with the remaining channels zero-filled—the protocol used for lead masking in Section 3.10—with MLII mapped to Lead II and no retraining or threshold adjustment. Window labels follow each database’s rhythm annotations, excluding windows that span a rhythm transition or are annotated as atrial flutter.

A dedicated dataloader per dataset harmonizes file formats and maps native rhythm labels to our binary AFib/non-AFib schema. All signals were then processed identically:

- **Resampling**—all recordings were resampled (or kept) at 500 Hz.
- **Robust amplitude normalization**—the 1st and 99th percentiles of each recording were computed jointly over all leads and time points, the signal clipped to that range and mapped linearly to [−1,1]. Percentiles rather than extrema make the scaling insensitive to electrode spikes and saturation, and pooling across leads preserves the relative inter-lead amplitude that carries diagnostic information. The identical routine is applied at inference time, so no training-to-serving discrepancy arises.
- **Fixed-length framing**—signals were zero-padded or truncated to 5000 samples (≈10 s) to enable mini-batch training.

Augmentations (training splits only): A single stochastic transformation was sampled per mini-batch from the list below; all augmentations preserve class membership.

- **Temporal transforms:** random circular phase shift; variable-length leading/trailing zero shifts.
- **Morphological noise:** baseline drift (linear), baseline wander (low-frequency sinusoids), EMG-like muscle artifacts (20– 100 Hz band-passed Gaussian noise).
- **Channel manipulations:** accidental lead reversal, random lead swapping, electrode contact artifacts (spikes, drop-outs, steps and jitter).
- **Spectral smoothing:** FFT low-pass ($f_{c}=40\mathrm{Hz}$, Hann transition) to simulate analogue antialias filtering.
- **Spatial masking:** per-sample binary lead mask to mimic missing channels (used later by the interpretability head).

All transformations were implemented on-the-fly to avoid duplicating data on disks and were disabled for validation, internal testing, and all external evaluations.

### 2.2. Model Architecture

**Figure 1:**
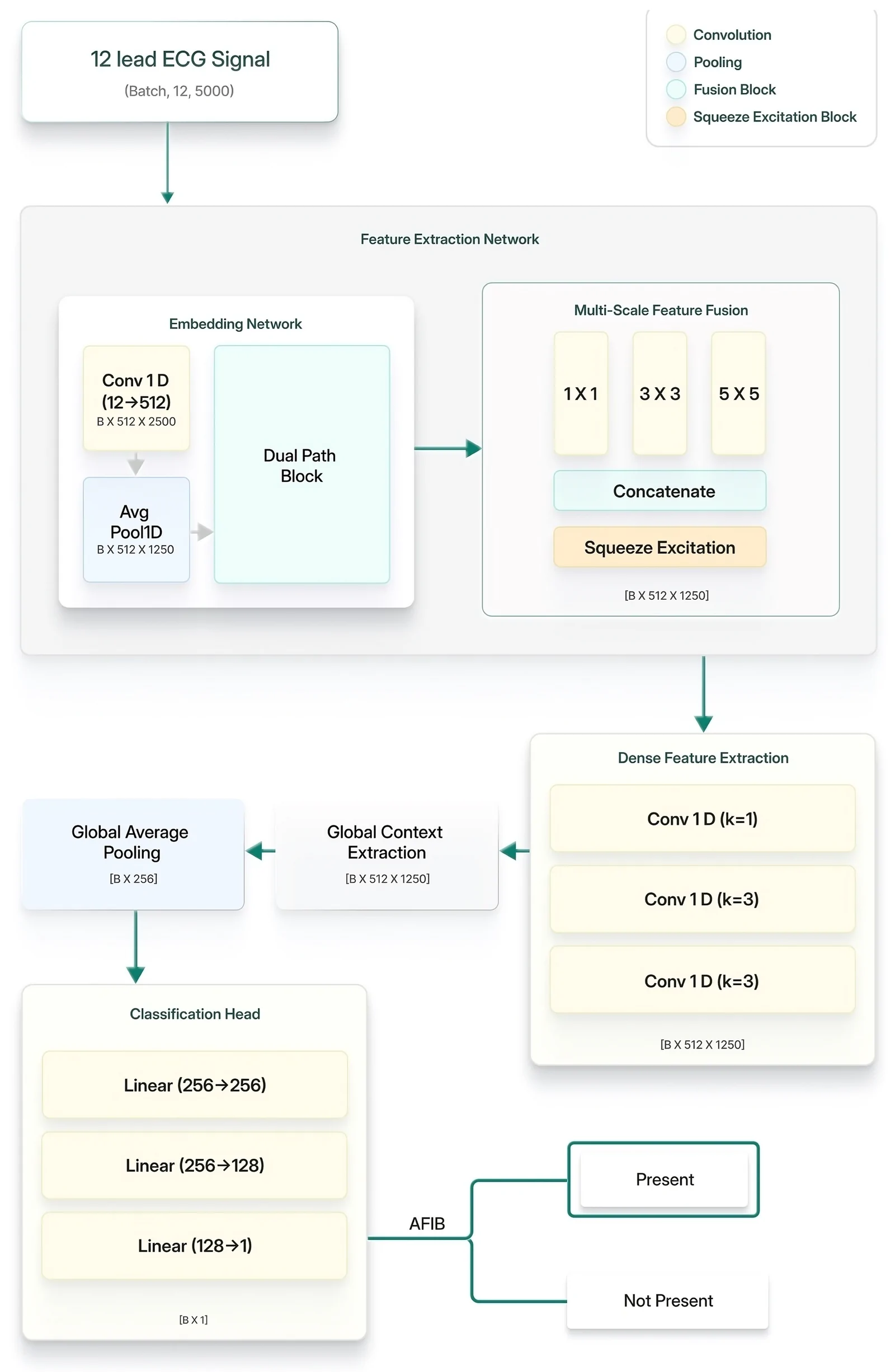
Overall model architecture showing the three main components: embedding network, feature extraction network, and classification head for AFib detection from 12-lead ECG signals.

We developed a deep learning architecture for automated atrial fibrillation (AFib) detection from 12-lead ECG signals. The architecture leverages the power of convolutional neural networks (CNNs) to extract and analyze temporal and spatial features from ECG signals. The proposed ECG classification model consists of three main blocks: feature extraction network, dense feature extraction, and classification head. Each block is designed to progressively extract and refine the features of the raw ECG signals for accurate binary classification, as illustrated in Figure 1.

#### 2.2.1. Feature Extraction Network

The feature extraction network transforms raw ECG signals into multi-scale representations through two sub-modules: an embedding network and multi-scale feature fusion.

##### Embedding Network

The 12 × 5000 input passes through a 1-D convolution (kernel 7, stride 2 and padding 3) to give a 512 × 2500 output, followed by batch normalization, ReLU and average pooling (kernel 3, stride 2 and padding 1) that downsamples to 512 × 1250.

The core component is the dual path block, comprising a residual path, a dense connection path and a squeeze-and-excitation mechanism. The residual path stacks two convolutional layers with wide kernels (size 15) to capture long-range temporal dependencies; the dense connection path uses 1 × 1 convolutions for efficient feature transformation.

The squeeze-and-excitation (SE) block is a channel-wise attention mechanism that adaptively recalibrates channel responses, improving feature selectivity at negligible cost.

##### Multi-Scale Feature Fusion

Three parallel convolutional branches with kernel sizes 1, 3 and 5 capture contextual information at different granularities: cross-channel interactions, short-term temporal patterns, and longer-range dependencies, respectively. Their outputs are concatenated, passed through a squeeze-and-excitation mechanism and fused back to the original channel dimension with a residual connection.

#### 2.2.2. Dense Feature Extraction

The dense feature extraction block refines the multi-scale features in two sequential stages.

##### Convolution

Batch normalization is applied to the embeddings, followed by convolutional layers that progressively refine them:

- The initial 1 × 1 convolutional layer reduces the dimensionality to 512 channels;
- Two sequential 3 × 3 convolutional layers with padding capture local temporal patterns;
- Batch normalization and ReLU activation are applied after each convolution;
- The final batch normalization ensures stable feature distributions.

##### Global Context Extraction

A 1 × 1 convolution reduces dimensionality to 256 channels, followed by batch normalization, ReLU and a squeeze-and-excitation block. This yields a compact global representation capturing temporal and contextual patterns across the entire ECG sequence, which the classification head consumes.

#### 2.2.3. Classification Head

The classification head transforms the global context features into binary classification outputs through a multi-layer neural network architecture. The process begins with adaptive average pooling that converts the temporal features into a fixed-size representation regardless of input sequence length.

The multi-layer classifier consists of three sequential linear transformations:

- The first linear layer projects the input features to 256 dimensions, followed by batch normalization and ReLU activation;
- The dropout layer with a rate of 0.4 provides regularization to prevent overfitting;
- The second linear layer reduces dimensionality to 128 features, followed by batch normalization, ReLU activation and dropout with a rate of 0.3;
- The final linear layer outputs a single logit for binary classification.

The entire architecture is trained end-to-end using binary cross entropy loss with class weighting to handle potential class imbalance in ECG rhythm classification tasks.

### 2.3. Training Pipeline

#### 2.3.1. Optimization Strategy

The model had random weight initialization and was trained using the AdamW optimizer with differential learning rates applied to various model components. The embedding network was initialized with a learning rate of $1\times 10^{-5}$, while the classifier components utilized a learning rate of $1\times 10^{-4}$. Weight decay was set to $1\times 10^{-4}$ to regularize model parameters. Gradient clipping was applied with a parameter of 1.0 to prevent gradient explosion.

#### 2.3.2. Learning Rate Scheduling

A cosine-annealing learning rate schedule was applied, stepped once per epoch, with a cycle length of $T_{\max}=20$ epochs and a minimum learning rate of $1\times 10^{-6}$. Within each cycle, the learning rate decays from its initial value to this minimum following a cosine curve and is then restored, so the schedule repeats across the training budget rather than decaying monotonically. Both learning-rate groups defined in Section 2 are annealed by the same schedule.

#### 2.3.3. Handling Class Imbalance

To mitigate the bias introduced by an uneven class distribution, we incorporate a class-weighted loss. Specifically, for binary classification, we compute a weight for the positive class as

(1) $$w_{+}\;=\;\frac{1}{f^{\alpha }}\;,\;f\;=\;\frac{N_{+}}{N_{+}+N_{-}}$$

where $N_{+}$ and $N_{-}$ denote the numbers of positive and negative samples in the training set, respectively; *f* is the empirical positive-class frequency; and α is a scaling exponent, set to α=0.5 in our experiments so that the positive class is up-weighted by $1/\sqrt{f}$ rather than by the full inverse frequency. The negative-class weight is taken as $w_{-}=1$. For the HDXML training split (7672 AFib of 53,945 records, f=0.142), this gives $w_{+}=2.65$. The square-root scaling emphasizes rare events while avoiding the excessive gradient weighting that a full 1/f factor would introduce at this class ratio and prevents the model from trivially predicting the majority class.

#### 2.3.4. Training Protocol and Reproducibility

Table 3 reports the complete training configuration used to produce the HDXML-only model evaluated throughout this work. The model was implemented in Python 3.10 with PyTorch 2.5.1.

The HDXML corpus was partitioned at the record level into 53,945 training and 13,487 validation records (an 80/20 split) using a fixed random seed of 42. No HDXML test partition was held out; all performances reported in this study are therefore measured on the external cohorts described in Section 2.1. Model selection was performed exclusively on the HDXML validation split: checkpoints were ranked by validation loss, and the epoch-57 checkpoint, which attained the minimum, is the one reported here. No early stopping was applied: training ran to a fixed budget of 200 epochs, and model selection was governed solely by the validation-loss checkpointing criterion described above.

The classification threshold was fixed a priori at τ=0.5, the default decision boundary for a sigmoid output, and was not tuned on any dataset. All external results in this study use that value. The multi-dataset model of Experiment 1 was evaluated at τ=0.7, chosen to favor specificity in a continuous-monitoring context; neither value was derived from an optimization procedure. Section 3.6 reports how the results vary with τ.

No external dataset was used at any stage for hyperparameter selection, early stopping, model selection or threshold calibration. Each external cohort was evaluated once, using the selected checkpoint, with no dataset-specific adjustment, retraining or re-calibration of any kind.

Training used 16-bit mixed precision, and deterministic kernel selection was not enforced.

### 2.4. Statistical Analysis

Point estimates are reported with 95% confidence intervals obtained by non-parametric bootstrap with 2000 resamples and percentile intervals. Because several cohorts contain multiple recordings per patient, or multiple analysis windows per recording, resampling was performed at the level of the independent unit rather than the individual prediction: clusters were drawn with replacement and all observations belonging to a drawn cluster were retained. Clusters were stratified by whether they contained a positive case, preserving both cluster count and class balance across resamples. The resampling unit is the patient for MIMIC-IV, CODE-15, SPH and CPSC 2021; the subject for MIT-BIH; and the record for PhysioNet/CinC 2021. Per-cohort cluster counts are given in Supplementary Table S3. Treating every prediction as independent would materially understate uncertainty in the ambulatory cohorts, where 173,630 and 8477 analysis windows derive from only 105 patients and 47 subjects, respectively. For CPSC 2021, the record identifiers encode the subject, and the 1435 evaluated recordings originate from 105 patients; resampling by recording rather than by patient would understate the interval by roughly a factor of two.

We deliberately do not report significance tests between cohorts. The cohorts are different populations with different AFib prevalences and acquisition conditions, so a *p*-value comparing them would not be interpretable. For the same reason, we do not test our results against published values from other studies, for which per-record predictions are unavailable. Paired significance testing is reported only where a single test set is held fixed, namely the lead-masking comparisons in Section 3.10.

### 2.5. Experiments Performed

Five experimental protocols were conducted to evaluate model performance and generalization:

1. **Multi-dataset training**: a single network was trained on the union of CODE-15, Chapman–Shaoxing, SPH, MedalCare-XL, the PhysioNet/CinC 2020 challenge set and PhysioNet/CinC 2021 (237,921 training, 29,740 validation and 29,740 test records) and then evaluated on the held-out internal split and on the unseen MIMIC-IV dataset (800,034 records). The cohorts identified as “Chapman–Shaoxing” and “Chapman-10k” in the submitted manuscript are, respectively, the SPH database and the Chapman–Shaoxing collection; see Section 2.1.
2. **P-wave mask augmented training**: AFib is characterized by irregularly irregular R–R intervals and absent P waves. A two-stage pipeline segmented P-wave regions with a compact 1-D U-Net, and an attention module directed the classifier towards those masks. The data are as in the multi-dataset experiment, with P-wave masks added.
3. **Vectorcardiogram (VCG)-based training**: the 12-lead input was transformed into a 3-lead VCG via the Kors transformation, with all subsequent preprocessing in the VCG domain and the same classifier architecture.
4. **HDXML-only training**: the network was trained exclusively on HDXML (67,432 ECGs; 9628 AFib, 57,804 non-AFib) and tested on every remaining dataset, quantifying cross-dataset generalizability from a single source.
5. **Variable-lead evaluation**: robustness under reduced lead availability, representative of ambulatory monitors, wearables and electrode dropout, was assessed with the same weights and no fine-tuning under two complementary masking protocols.
  - *Phase 1—Lead II only*: all 11 leads except Lead II were zeroed, simulating a single-lead ambulatory recording.
  - *Phase 2—Random masking*: 3, 6 or 10 leads were randomly masked (leaving 9, 6 or 2 active leads, respectively); each condition was repeated over 5 independent random seeds, and results are reported as mean ± standard deviation.
  - *Optional configurations*: fixed anatomical subsets were also assessed: limb leads only (I, II, III, aVR, aVL and aVF), precordial leads only (V1–V6) and four individual single leads (I, II, III and aVF).
  All evaluations used the identical model checkpoint; no retraining or threshold adjustment was performed.

## 3. Results

### 3.1. Secondary Experiments

Three protocols preceded the single-source model and are reported in full in Supplementary Section S1. Briefly: a network trained on the union of six datasets reached 97.5% accuracy on its held-out split and 95.7% accuracy (70.4% sensitivity and 98.5% specificity) on MIMIC-IV; directing the classifier at segmented P-wave regions did not improve on it (95.4% accuracy on MIMIC-IV); and a vectorcardiogram formulation via the Kors transformation improved robustness to noisy recordings but reduced sensitivity across all datasets. None outperformed the HDXML-only model reported below, which is why that model was carried forward. All metrics reported for these three experiments are consolidated in Supplementary Table S1; note that they were evaluated at τ=0.7 and so are not directly comparable with the τ=0.5 results below.

### 3.2. HDXML-Only Training: Cross-Dataset Generalization

The HDXML-only training experiment demonstrates strong cross-dataset generalization. As shown in Table 4, ROC–AUC exceeded 0.95 on every one of the six independently sourced external cohorts, ranging from 0.960 (95% CI 0.956–0.963) on CODE-15 to 0.999 (0.998–0.999) on SPH. Confidence intervals are narrow on the large resting-ECG cohorts and wider on the ambulatory cohorts, where the resampling unit is the recording or the subject rather than the individual window.

**Figure 2:**
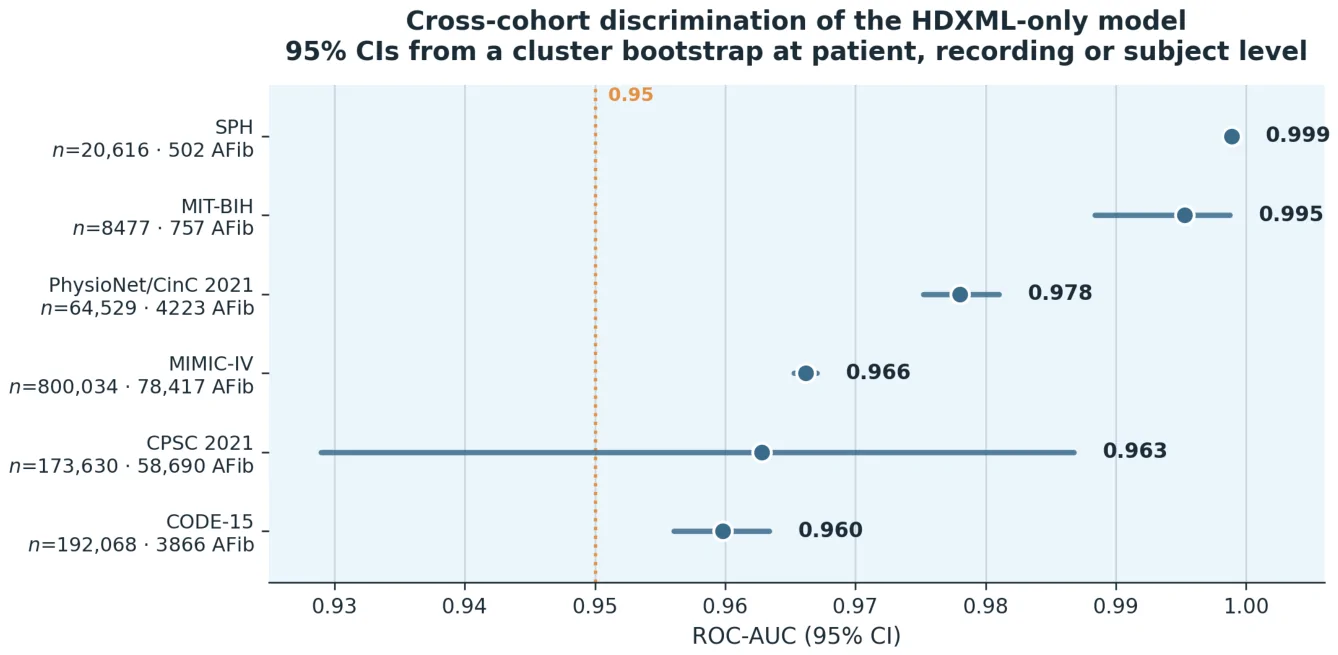
Cross-cohort discrimination of the HDXML-only model. Points are ROC–AUC and bars are 95% confidence intervals from a cluster bootstrap, with the resampling unit of Supplementary Table S3. The dotted line marks 0.95; every point estimate exceeds it, and the CPSC 2021 interval extends below it because its 173,630 analysis windows derive from only 105 patients.

**Figure 3:**
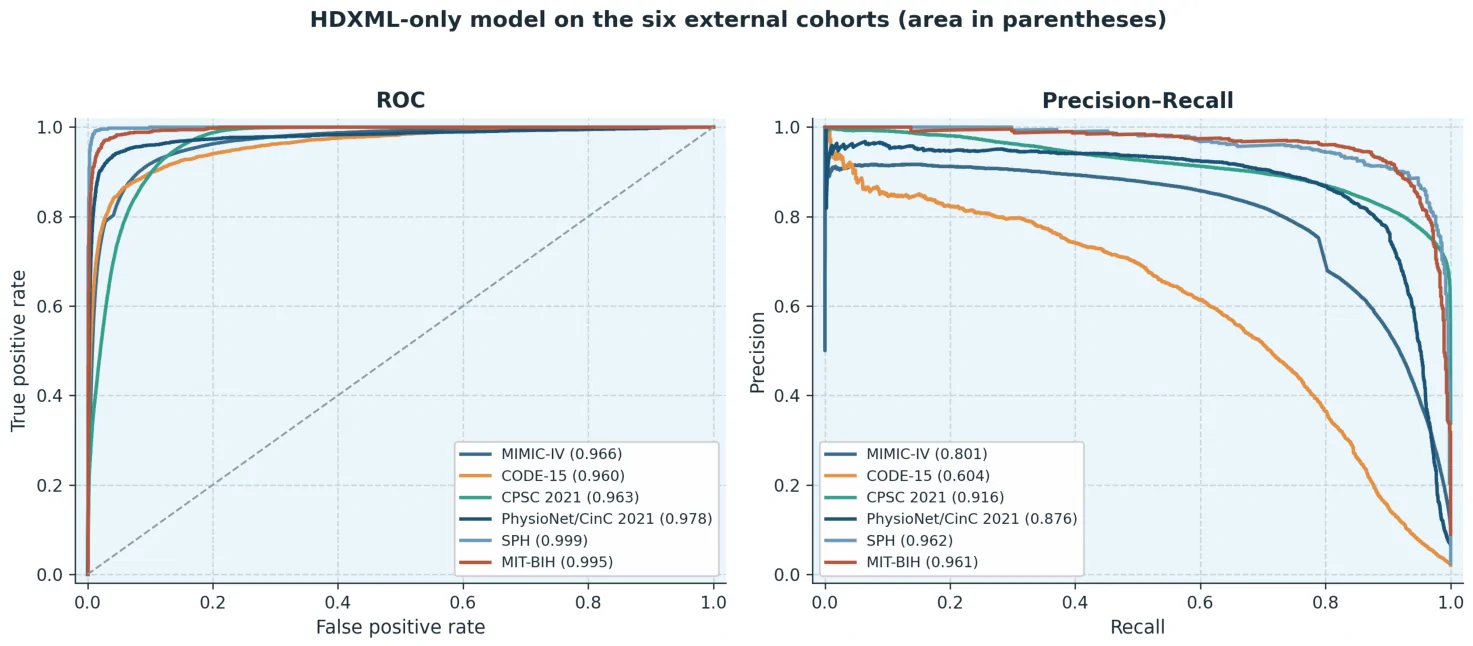
ROC (**left**) and precision–recall (**right**) curves for the HDXML-only model on the six external cohorts, from the same predictions as Table 4. The dashed diagonal marks chance performance. The precision–recall panel separates the cohorts far more because precision depends on prevalence.

Figure 2 shows the six cohorts with their confidence intervals, and Figure 3 the corresponding ROC and precision–recall curves.

Performance was transferred to ambulatory two-lead Holter recordings without retraining: ROC–AUC 0.963 (0.929–0.987) on CPSC 2021 and 0.995 (0.988–0.999) on MIT-BIH. Within the CPSC 2021 records annotated as paroxysmal atrial fibrillation, where episodes begin and end inside a single continuous recording, the model reached ROC–AUC 0.999 with 97.1% sensitivity and 99.7% specificity, indicating that it discriminates rhythm transitions within a patient rather than patient-level characteristics.

Performance variability was observed on CODE-15, where sensitivity decreased (58%) despite high specificity (>99%). This likely reflects domain shift or label heterogeneity within CODE-15 rather than model instability, as discriminative capacity (ROC–AUC 0.96) remained high.

In general, these findings indicate that the features learned from a single curated training source are effectively transferred across heterogeneous external datasets without retraining.

### 3.3. Saliency Map Analysis

**Figure 4:**
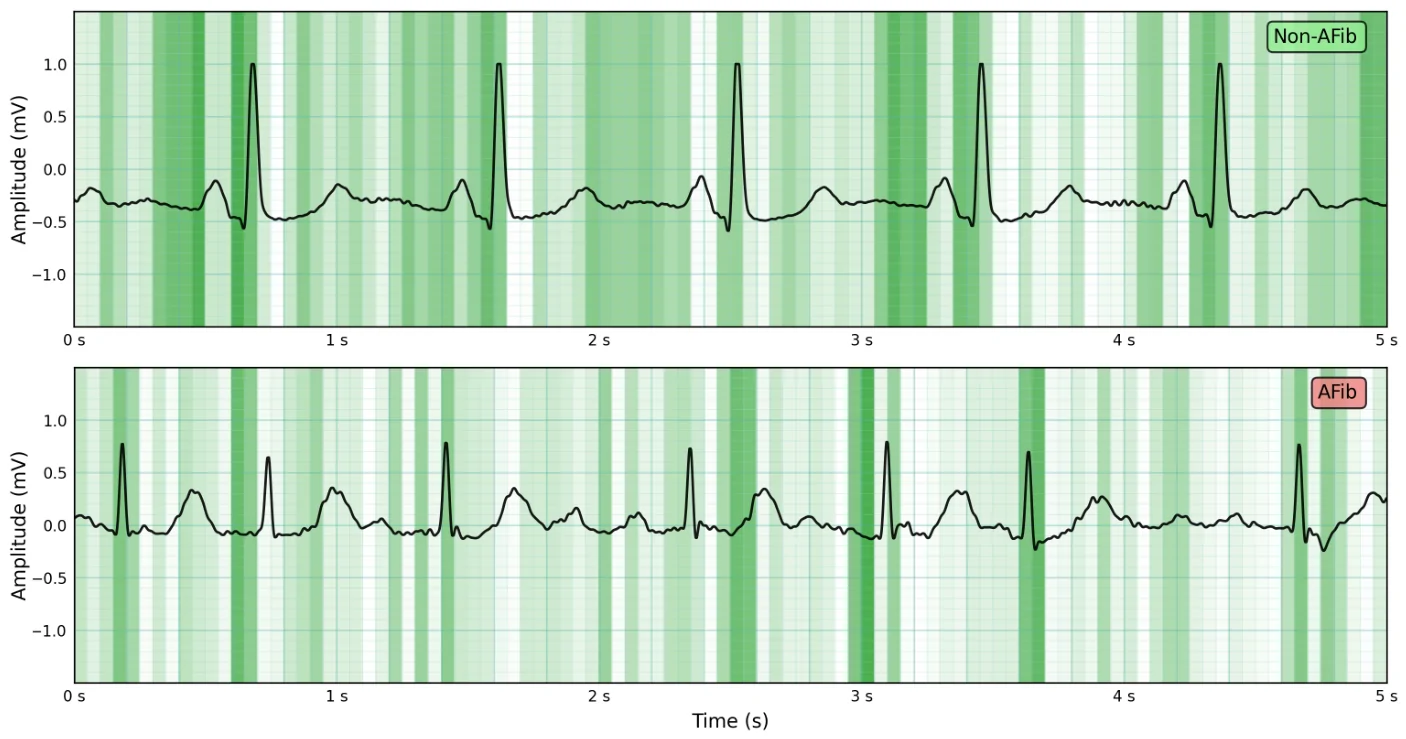
Occlusion sensitivity maps for a representative non-AFib (**top**) and AFib (**bottom**) recording. Shading is the change in predicted probability when a sliding window at that position is zeroed, normalized to [0,1] per recording. Darker green indicates a larger change.

Figure 4 overlays an occlusion sensitivity map on two representative recordings, one predicted non-AFib and one predicted AFib, each shown over a 5 s window of Lead II. A sliding window is zeroed at each position in turn, and the resulting change in the predicted probability is recorded, so intensity measures how much the decision moves when that part of the trace is removed; values are normalized to [0,1] within each recording.

The two panels differ in structure. On the non-AFib recording, sensitivity is concentrated in narrow bands aligned with the regular QRS complexes and the consistent intervals separating them. On the AFib recording, it is distributed more diffusely across the segments between R peaks—the region in which the P wave is absent and fibrillatory activity appears, and whose varying length constitutes the R–R irregularity. This is consistent with the two clinical criteria for AFib.

Attribution maps of this kind show where a model looks; they do not establish that the decision depends on what is there. We therefore treat this analysis as descriptive and quantify the dependence in Section 3.4.

### 3.4. Causal Evidence from Input Perturbation

Saliency maps indicate where a model attends, but attention is correlational. To test whether the model’s decisions depend causally on the features that define AFib clinically, we perturbed the input and measured the effect.

**Region occlusion.** Beats were located by R-peak detection on Lead II using NeuroKit2 [32], and the interval containing the P wave, the QRS complex or the T wave was occluded across all twelve leads. Intervals follow fixed physiological offsets from each R peak rather than a learned delineator: in AFib, the P wave is absent by definition, so the interval in which it would fall is well defined for every beat and is where fibrillatory activity appears. The decisive comparison is against a control occluding the same total duration at random positions, which separates a region-specific effect from the effect of removing a signal.

**Figure 5:**
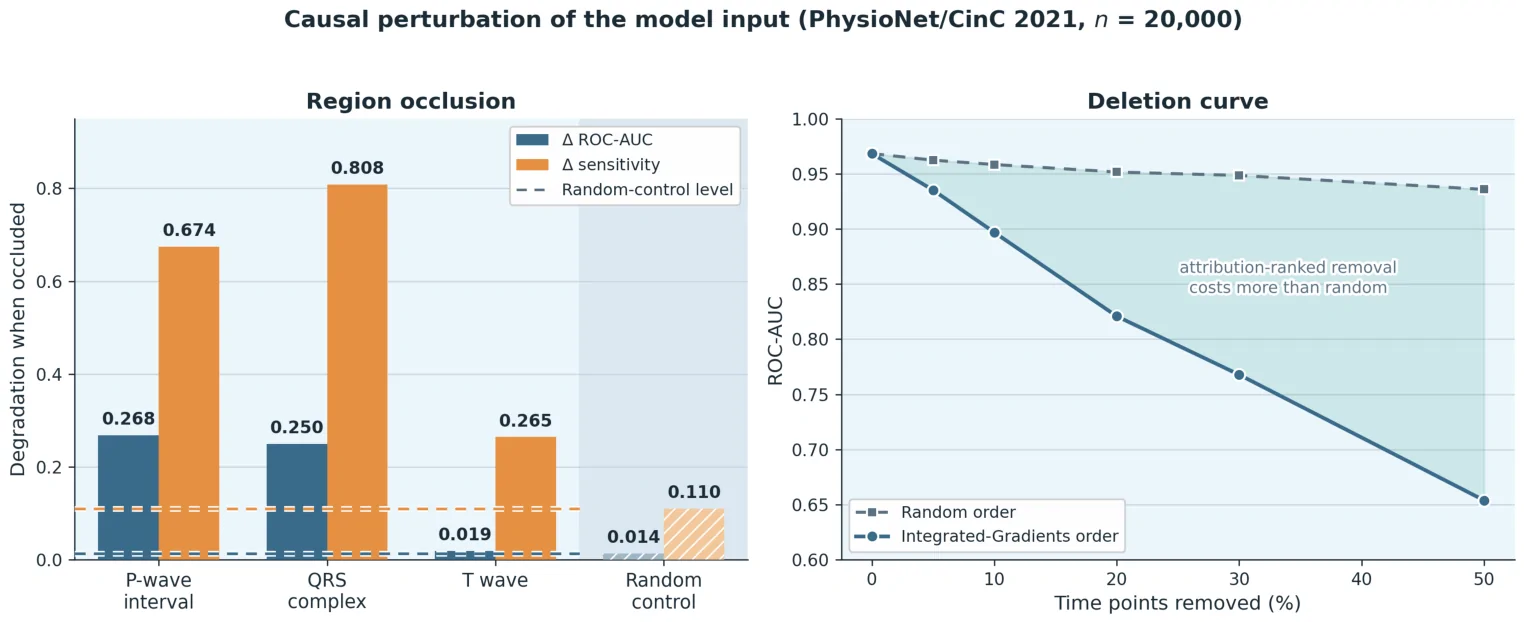
(**left**) Loss in ROC–AUC and in sensitivity when each region is occluded across all twelve leads. The random control (hatched) occludes the same total duration at random positions, so a bar’s height above its own dashed line is the region-specific effect rather than the cost of removing signal. The dashed lines mark the random-control level of each metric (blue, change in ROC–AUC; orange, change in sensitivity), and the shaded column holds the random-control bars. (**right**) ROC–AUC as time points are removed in order of Integrated Gradients’ attribution against random order; the shaded band is the excess degradation attributable to the ranking.

Table 5 and Figure 5 report the result. Occluding the P-wave interval reduces ROC–AUC by 0.268 and sensitivity by 0.674, against 0.014 and 0.110 for the duration-matched random control—a region-specific effect roughly twenty times larger than removing an equal amount of signals elsewhere. Occluding the QRS complex is comparable (0.250 and 0.808), consistent with the clinical definition of AFib, since removing QRS complexes destroys the R–R interval irregularity that is its second diagnostic criterion. The T wave, not a diagnostic criterion for AFib, contributes far less (0.019).

**Deletion curve.** The occlusion analysis above requires the regions of interest to be named in advance. As a second region-free test, we let the model itself rank the input.

Integrated Gradients [33] attributes the output to each input sample by accumulating gradients along the straight path from a baseline $x^{\prime }$ to the recording *x*. For model *F*, the attribution of sample *i* is

(2) $${\mathrm{IG}}_{i}\left(x\right)\;=\;\left(x_{i}-x_{i}^{\prime }\right)\int_{0}^{1}\frac{\partial F\;\left(x^{\prime }+\alpha \;\left(x-x^{\prime }\right)\right)}{\partial x_{i}}\;d\alpha ,$$

evaluated with a 16-step Riemann sum in Captum [34]. We use the all-zero baseline, which is also what deletion writes into a sample, so attribution and intervention are defined consistently; attributions were taken in absolute values and summed across the twelve leads to give one score per time point.

Time points were then deleted in descending order of that score and the model re-scored against a random-order control. If the attributions locate information the model uses, removing the top-ranked points must cost more than removing the same number at random. Removing the 10% most attributed costs 0.072 ROC–AUC against 0.010 at random; at 50%, 0.314 against 0.032. The area under the deletion curve is 0.401 for the attribution order against 0.475 at random, where a lower value means the ranking found more of what mattered. This supports the interpretability analysis of Section 3.3.

**Lead redundancy.** Occluding any single lead while retaining the other eleven changes ROC–AUC by at most 0.0019, and by 0.0016 for Lead II. With the full electrode set, the model draws on substantially redundant information, which is consistent with the stability observed under random masking in Section 3.10; Lead II’s individual strength emerges when it is the only lead available rather than when the others are present.

### 3.5. Comparison with Compact Architectures

To establish whether the generalization reported here depends on our particular architecture, we trained 1-D adaptations of three networks designed for constrained hardware—SqueezeNet, MobileNet and a narrow ResNet—on HDXML under the identical protocol of Table 3 and evaluated them on the same records (Table 6). Latency there is single-threaded FP32 inference at batch size 1 on the training host, a dual-socket Intel Xeon Platinum 8481C at 2.70 GHz with 104 cores running PyTorch 2.5.1, and is reported for comparison between architectures on common hardware rather than as a deployment figure.

**The training recipe transfers across architectures.** All four networks reach comparable discrimination on all six cohorts: our architecture attains the higher ROC–AUC on three and a compact baseline on the other three, with margins of 0.0028 or less in our favor and up to 0.0302 in theirs. Compact designs additionally offer a lower computational profile, with SqueezeNet-1D using 15.7 times fewer parameters, 56 times fewer multiply–accumulate operations and 37.6 times less single-threaded CPU time. That four independently designed networks all generalize to the same six cohorts, from one training source and with no per-cohort tuning, indicates that the generalization reported here is a property of the training data and protocol rather than of one particular network. This is consistent with prior comparisons of compact backbones for ECG, in which a sub-1M-parameter SqueezeNet was competitive with substantially larger networks [35].
**The accuracy differences should not be read as architectural.** Each architecture was trained once. To calibrate what a single run can resolve, our own architecture trained twice under near-identical protocols—the released checkpoint and the retrained arm of Section 3.7—differs from itself by up to 0.0220 ROC–AUC on MIT-BIH and 0.0079 on CODE-15. Every architectural difference in Table 6 except CPSC 2021 is smaller than that same-architecture variability, and the CPSC 2021 difference is itself about half the width of that cohort’s confidence interval. These architectures are therefore statistically indistinguishable at this evidence level, and we make no claim that any is more accurate than another; establishing a genuine architectural difference would require multiple runs per architecture, which we identify as a limitation.
**Practical implication.** Parameter and operation counts are exact and latency is repeatable, so the computational comparison carries no such uncertainty, and it offers a useful degree of freedom: where computational budget is binding, a network an order of magnitude smaller can be substituted while retaining the cross-cohort behavior demonstrated here. The central result of this study—that a single, modest, carefully curated training source supports generalization at this scale—is correspondingly robust, holding across architectures spanning 0.90 M to 14.1 M parameters.

### 3.6. Probability Calibration and Threshold Sensitivity

Because every reported sensitivity and specificity depends on the decision threshold, we report how well the model’s outputs behave as probabilities and how far the results move when τ is varied.

**Figure 6:**
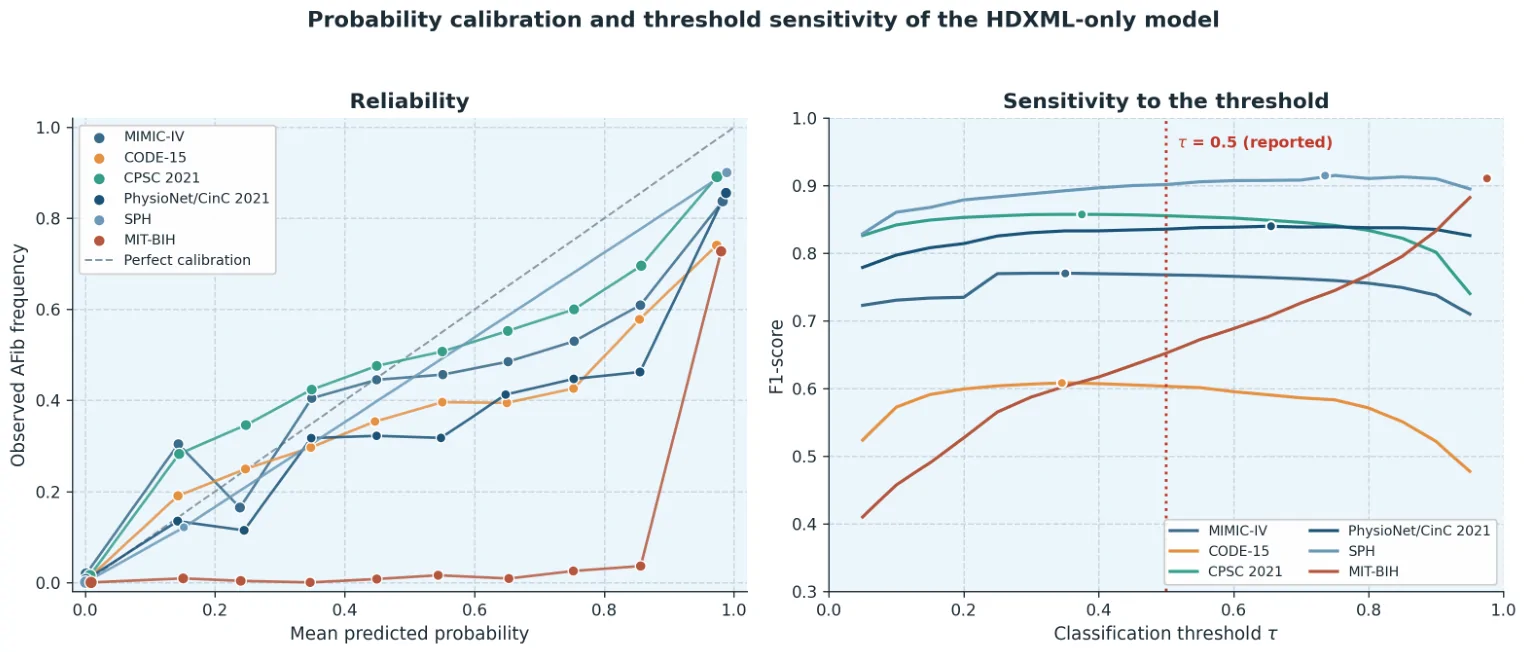
(**left**) Reliability diagram: mean predicted probability against observed AFib frequency in ten equal-width bins, marker area scaling with the logarithm of bin count; points on the dashed diagonal are calibrated. (**right**) F1 against classification threshold, with each cohort’s optimum (dots) and the reported τ=0.5 (dotted line) marked.

**Calibration.** The model is well calibrated on five of the six cohorts without post hoc adjustment: mean predicted probability tracks observed prevalence closely—0.097 against 0.098 on MIMIC-IV and 0.021 against 0.020 on CODE-15—with Brier scores from 0.004 to 0.077 and expected calibration error below 0.05 (Table 7 and Figure 6). This bears on the threshold: when probabilities are calibrated, τ=0.5 is the Bayes-optimal cut under equal misclassification costs, making the a priori value principled rather than conventional.

MIT-BIH is the exception: expected calibration error 0.108, with mean predicted probability 0.197 against an observed rate of 0.089. This over-confidence is consistent with its acquisition—analog Holter tape from 1975 to 1979, two leads and amplitudes unlike anything in the training corpus—and affects the probabilities rather than the ranking, since its discrimination is the highest of any cohort.

**Sensitivity to τ.**Table 7 reports sensitivity and specificity at τ∈{0.3,0.5,0.7}, and Figure 6 shows F1 across the full range. The curves are flat around 0.5 on every cohort except MIT-BIH: moving from 0.3 to 0.7 changes MIMIC-IV sensitivity from 0.780 to 0.723 and specificity from 0.974 to 0.981. This follows from the output distribution, in which 76% to 99% of predictions fall outside [0.05,0.95].
**Why no optimal threshold is selected.** The F1-maximizing threshold ranges from 0.345 on CODE-15 to 0.975 on MIT-BIH, and the Youden-optimal threshold spans a wider range still. This is expected, since precision depends on prevalence and prevalence varies from 2.0% to 33.8% here: an F1-optimal threshold describes the cohort as much as the model. Selecting one value would privilege a single population, and selecting it on the evaluation data would compromise the independence of the external validation. The mean F1 forgone by retaining τ=0.5 is 0.048, most of it attributable to MIT-BIH. A deployment should choose its operating point against the prevalence and cost structure of its target population rather than adopt a value tuned on retrospective cohorts.

### 3.7. Component Ablation

Ten configurations were trained from scratch on HDXML under the protocol of Section 2.3.4—identical seed, split, optimizer, schedule, augmentation and 80-epoch budget—so that the architecture is the only variable; the complete architecture was retrained rather than reused, so all arms are comparable. Five add a single component to a baseline consisting of the convolutional path alone, and five remove one from the complete architecture (Table 8).

**Discrimination on 12-lead input is insensitive to most components.** Excluding the dense feature stack, all configurations fall within 0.0044 ROC–AUC of one another on PhysioNet/CinC 2021, a range narrower than most individual confidence intervals. The baseline CNN, using 29% fewer parameters and 24% fewer operations, attains 0.9412 against 0.9393 for the complete architecture (p=0.14 after correction) and 0.9692 against 0.9519 on CODE-15. Several arms differ at nominal significance, but with effect sizes of order 0.003 ROC–AUC that reach significance only because the cohort contains 70,602 records. The dense feature stack is the exception: removing it costs 0.0159 ROC–AUC on PhysioNet/CinC 2021 and 0.0117 on CODE-15, the only ablation whose effect is both unambiguous (p<0.001) and large relative to between-cohort differences.
**Squeeze–excitation contributes specifically under reduced-lead input.** The 12-lead comparison finds no effect of squeeze–excitation (Δ = 0.0008, *p* = 0.57), which alone would suggest the mechanism is inert; evaluating the same arms under lead masking shows otherwise. Table 9 reports two contrasts differing only in the presence of squeeze–excitation—the complete architecture against the same network with it removed from all three placements, and the baseline CNN with and without it in the convolutional block—and both show the same pattern. The benefit is absent at 12 leads; small and inconsistent at six; and reaches +0.0175 and +0.0202 ROC–AUC, respectively, on Lead II alone, all with *p* < 0.001 after correction. In the second contrast, it is marginally harmful at 12 leads (−0.0026) and clearly beneficial at one.

This is direct evidence for the mechanism proposed in Section 3.10: channel attention offers little when every lead is present and informative, and becomes useful precisely when the network must redistribute representational weight across fewer channels. A 12-lead-only ablation would have reached the opposite conclusion, indicating that components should be evaluated under the conditions in which they are claimed to operate. The same mechanism has been arrived at independently: LightX3ECG aggregates three leads through a lead-wise attention module for precisely this reason [36].

### 3.8. Label Coding in the Ningbo Subset of PhysioNet/CinC 2021

The Ningbo database, which contributes 27,865 of the 70,602 PhysioNet/CinC 2021 records, assigns atrial flutter to 7615 records and atrial fibrillation to none—implausible in a clinical corpus of that size, since fibrillation is substantially more common than flutter. On those flutter-coded records, the model’s predicted probabilities behave as they do on confirmed AFib elsewhere (median 0.992) and unlike its behavior on flutter in other databases of the same release (median 0.199 on Chapman–Shaoxing and 0.004 on PTB-XL), and they account for 88.4% of all false positives on the cohort under the original labels.

We therefore exclude the 6073 flutter-coded Ningbo records in our evaluation split—the conservative option, removing ambiguous labels rather than reassigning them. Discrimination is essentially unchanged across all four policies we examined, so the conclusion does not depend on the choice; Supplementary Section S6 and Table S6 give the full audit.

### 3.9. Error Structure and Predictive Values

Errors are asymmetric and cohort-dependent. On CODE-15, the model is highly specific (99.3%) but detects 58.3% of AFib cases; on MIT-BIH, it detects 99.2% at a specificity of 89.7%. Positive predictive value therefore varies with prevalence, which ranges from 2.0% on CODE-15 to 33.8% on CPSC 2021, and observed PPVs are not directly comparable between cohorts.

Standardizing predictive values to fixed reference prevalences shows that at the low prevalence typical of community screening, specificity rather than sensitivity dominates achievable PPV: at π=2%, the CODE-15 operating point yields a PPV of 0.63 against 0.53 for PhysioNet/CinC 2021, despite the latter’s far higher sensitivity. A screening deployment would therefore need to select its operating point against the prevalence of the intended population rather than against discrimination alone. Full confusion matrices and the standardized predictive values are in Supplementary Section S7, Tables S7 and S8.

### 3.10. Variable-Lead Evaluation: Robustness Under Reduced Lead Availability

**Rationale and hypothesis.** Real-world AFib monitoring spans a wide range of hardware, from standard 12-lead recorders to single-lead wearables and ICU telemetry with intermittent electrode dropout. A deployable detector must therefore retain diagnostic utility when lead information is reduced. We hypothesized that the squeeze-and-excitation channel-attention mechanism would redistribute representational weight across whichever leads remain active, sustaining discriminative capacity without retraining. Section 3.7 tests that hypothesis directly.

PhysioNet/CinC 2021 was selected for this experiment. It is the largest multi-source resting cohort available to us, and the challenge from which it derives was itself concerned with reduced-lead electrocardiography, which makes the benchmark relevant to a community-defined problem rather than an ad hoc protocol. Every configuration is evaluated on the same 64,529 records with the same checkpoint, so configurations are directly comparable; missing leads are represented by zero-valued channels at inference, and no parameter or decision threshold was altered.

- **Statistical treatment.** The claim under test is that performance is retained under lead reduction. A non-significant difference would not establish that, since absence of evidence is not evidence of equivalence. We therefore pre-specified a non-inferiority margin of 0.02 ROC–AUC and test each configuration against the 12-lead baseline with a one-sided paired DeLong test; because every configuration is scored on the same records, the paired form applies. Two-sided tests are Holm-corrected across configurations.

#### 3.10.1. Random Lead Masking

Table 10 reports performance as the number of available leads falls from 12 to 9, 6 and 2. Discrimination degrades monotonically and slightly: ROC–AUC moves from 0.9780 at 12 leads to 0.9779±0.0006 at nine, 0.9773±0.0016 at six and 0.9625±0.0049 at two. The nine- and six-lead configurations are non-inferior at the 0.02 margin in all five seeds, and the two-lead configuration in four of five. Specificity is the more stable of the two operating characteristics, remaining above 0.97 throughout, while sensitivity falls from 0.880 to 0.805±0.017 at two leads.

Reduced-lead classification is itself established: the PhysioNet/CinC 2021 Challenge found that entries scored comparably on 2-lead and 12-lead hidden test data [26]. Those entries, and subsequent reduced-lead models [37,38], train a configuration per lead set; the experiment here instead holds one checkpoint fixed across all of them.

#### 3.10.2. Fixed Lead Subsets and Single-Lead Performance

Either electrode group alone is sufficient: the precordial subset reaches ROC–AUC 0.9755 and the limb subset 0.9721, both non-inferior to full 12-lead input. Supplementary Figures S3 and S4 compare the fixed configurations and summarize all ten conditions.

Among individual leads, the ordering is unambiguous. **Lead II is markedly the strongest** at ROC–AUC 0.9681 (95% CI 0.9654–0.9707) and is the only single lead non-inferior to the full recording at the pre-specified margin. Lead I follows at 0.9317, Lead aVF at 0.9112 and Lead III at 0.7786. This hierarchy is consistent with Lead II’s conventional role as the rhythm lead: its axis is closely aligned with the mean atrial depolarization vector, so it carries the largest projection of P-wave amplitude and of the RR irregularity characteristic of AFib. Lead III, oriented more laterally in the frontal plane, carries the weakest projection and performs correspondingly poorly, with sensitivity of 0.330.

An earlier version of this analysis, conducted on a 280-record subset of the PhysioNet/CinC 2020 challenge set containing only 17 AFib cases, placed Lead aVF ahead of Lead II. That ordering does not survive evaluation on a cohort two orders of magnitude larger, and the present result supersedes it.

Key findings.

1. **Stable performance under lead reduction:** ROC–AUC remains within 0.016 of the 12-lead baseline down to two randomly retained leads, and the nine-, six- and two-lead configurations are non-inferior at a pre-specified 0.02 margin.
2. **Single-lead feasibility is specific to Lead II:** Lead II alone attains ROC–AUC 0.9681 and is non-inferior to the full recording, whereas Leads I, aVF and III are not. Claims of single-lead viability should name the lead rather than treat single-lead input as one condition.
3. **A replicable benchmark:** to our knowledge, this is the first AFib study to report performance under progressive random lead masking with a fixed checkpoint on a cohort of this size, with non-inferiority testing against a pre-specified margin.

## 4. Discussion

### 4.1. Contributions and Comparative Performance

This study provides four principal contributions.

**(1)**  **Cross-site external validity.** Table 4 reports performance on six independently sourced unseen cohorts. Competing studies typically publish a single external test, if any, and rarely include ICU records. Only ECG-AI [39] evaluated on MIMIC-IV, reporting ROC–AUC 0.77 against our 0.966 (95% CI 0.965–0.967). Entries in Table 11 come from different populations and protocols and so provide context rather than a controlled comparison (Section 4.2).
**(2)**  **Specificity under covariate shift.** False positives drive alarm fatigue and unnecessary anticoagulation. Specificity remains ≥90% on all six cohorts and 99.3% on CODE-15, while positive predictive value, being prevalence-dependent, varies more widely: 0.850 on SPH and 0.824 on CPSC 2021 against 0.486 on MIT-BIH.
**(3)**  **Robustness to lead variability.** Systematic masking shows performance is stable under progressive lead reduction, supporting deployment in wearable and reduced-channel settings. To our knowledge, no prior AFib study has reported a comparable lead-masking benchmark without retraining.
**(4)**  **Faster-than-real-time CPU inference.** The network contains 14,072,321 parameters (14.1 M; 56.3 MB in FP32) and requires 16.7 GMACs per 10 s 12-lead segment, an arithmetic cost characteristic of maintaining full channel width at high temporal resolution rather than one optimized for constrained inference; Section 3.5 quantifies that trade-off against architectures designed for it, and the released checkpoint stores a further 2,888,704 parameters (2.9 M) in two modules the forward pass never references. Measured in a containerized cloud environment on an AMD EPYC 7B13 CPU (Zen 3; three vCPU requested, 15 GB memory limit, no GPU allocated; PyTorch 2.6.0 with oneDNN, batch size 1, FP32), median latency was 110 ms per segment with four intra-op threads and 410 ms single-threaded, of which preprocessing accounted for 1.7 ms, at a peak resident memory of 658 MB. Inference therefore runs at 24–91 times real time on commodity server CPUs without a GPU and without quantization, within a sub-gigabyte footprint—the property that matters for bedside or ambulatory use. Where computational budget is the binding constraint, Section 3.5 indicates a substantially smaller network would serve.

The HDXML-only experiment thus demonstrates that a single high-quality training source is sufficient to yield a detector that generalizes across cohorts, addressing prior work that either lacks rigorous external validation or relies on far larger models; Section 3.5 shows this property follows from the training data and protocol rather than from one particular architecture.

Its cross-cohort generalization makes this model a promising candidate for clinical evaluation, though the present study is retrospective and prospective validation would be required before clinical use.

### 4.2. Interpreting the Comparison with Prior Work

Table 11 collects reported results from recent AFib detection systems. These studies differ in the datasets they evaluate on; the number of leads and signal duration they consume; the sampling rates and preprocessing they apply; and the task they address, which ranges from screening for arrhythmia to diagnosis-quality detection and to risk prediction in patients currently in sinus rhythm. They also report different primary metrics. The table is therefore a contextual summary of the field rather than a controlled benchmark, and apparent differences between rows should not be read as evidence that one method outperforms another.

For the same reason, we report no significance tests against these studies: their per-record predictions are not available, so no paired comparison is possible, and an unpaired test across different populations would not be interpretable. The comparisons we do quantify are those in which a single test set is held fixed and only the model or its input varies.

### 4.3. Performance Analysis

Across the six external cohorts, the detector holds ROC–AUC above 0.95 without retraining or per-cohort tuning, which is the property this study set out to test. We do not claim precedence over published systems: as Section 4.2 sets out, the entries in Table 11 come from different populations and protocols, so they provide context rather than a ranking. The HDXML-only experiment yielded well-balanced metrics on every cohort except CODE-15, where sensitivity is low despite high discrimination.

### 4.4. Clinical Relevance

The occlusion sensitivity maps of Section 3.3 illustrate attention to clinically meaningful regions, and the perturbation analysis of Section 3.4 establishes the dependence quantitatively: removing the interval in which the P wave falls costs 0.268 ROC–AUC against 0.014 for a duration-matched random control. The model’s behavior is therefore aligned with the diagnostic criteria for AFib rather than with an incidental correlate. The high specificity achieved across all settings is also important in minimizing false alarms in clinical monitoring, a recognized barrier to adoption.

### 4.5. Robustness to Signal Quality

Variable-lead experiments confirm that the model maintains discriminative stability even when substantial channel information is removed. ROC–AUC remained above 0.97 under random masking conditions and above 0.94 for clinically relevant single-lead configurations (Lead II and Lead aVF). This robustness is critical for wearable and ambulatory systems where full 12-lead acquisition is rarely available and electrode dropout is common.

This augmentation strategy was designed to encourage rhythm-based rather than morphology-specific representations and to expose the model during training to the noise, drift and missing-channel conditions it meets in clinical ECGs; we did not ablate it, so its individual contribution is not quantified here.

### 4.6. Limitations

Performance variability across the CODE-15 dataset underscores the need for careful dataset-specific analysis. The variable-lead experiments are conducted on a single external cohort, PhysioNet/CinC 2021 (n=64,529); confirming them on an independently sourced cohort would strengthen the reduced-lead claim further. The squeeze–excitation attribution of Section 3.10 is supported by the ablation of Section 3.7, which finds the component inert on 12-lead input and significantly beneficial under lead reduction; that ablation also indicates that a simpler configuration suffices for 12-lead discrimination, with the full design contributing chiefly when leads are missing. The model has not yet been prospectively validated in real-world clinical workflows. Regarding cohort independence, the Chapman–Shaoxing collection and the PhysioNet/CinC 2020 challenge set are contained within PhysioNet/CinC 2021; we therefore report them separately in Table 2 rather than counting them as independent cohorts. Record identifiers differ between releases of the same underlying data, so containment was established from release provenance and identifier ranges rather than by name matching, and residual patient-level overlap between separately sourced cohorts cannot be formally excluded. The two ambulatory cohorts contribute many analysis windows per recording, and MIT-BIH in particular provides only seven unique patients with atrial fibrillation; confidence intervals for these cohorts are computed with the recording or subject as the resampling unit and are correspondingly wide. Each architecture in Section 3.5, including our own, was trained once; because two runs of our own architecture differ by up to 0.0220 ROC–AUC, that comparison can establish equivalence of accuracy but not an ordering, for which multiple runs per architecture would be required. Finally, the label coding of the Ningbo subset (Section 3.8) required a curation decision, though performance was insensitive to which policy was adopted.

## 5. Conclusions

We presented a compact 14M-parameter CNN for AFib detection from 12-lead ECGs. Two training protocols were compared, and the single-source protocol was carried forward:

1. **A single curated training source was sufficient.** A model trained exclusively on a single, meticulously curated HDXML corpus achieved ROC–AUC above 0.95 on six of the six independently sourced external test sets—often matching or surpassing far larger architectures—while retaining a very high mean ROC–AUC (0.960–0.999). Only the CODE-15 cohort exhibited a marked sensitivity drop, pointing to a domain mismatch rather than a weakness in the model itself.
2. **High specificity under covariate shift.** The HDXML-only detector attained specificity ≥90% on every external cohort, reaching 99.3% on CODE-15 and 98.4% on PhysioNet/CinC 2021. High specificity matters for hospital monitors and wearables, where false alarms drive alarm fatigue and unnecessary anticoagulation, though the positive predictive values reported in Table 4 show that performance at a given operating point remains prevalence-dependent.
3. **Physiologically aligned and computationally efficient.** Occlusion sensitivity maps localize the model’s attention to the beat complexes and the intervals between them, and input perturbation confirms the decision depends on the two regions that define AFib clinically: occluding the P-wave interval or the QRS complex costs 0.268 and 0.250 ROC–AUC, respectively, against 0.014 for a duration-matched random control. The model occupies 56.3 MB in FP32 with a 658 MB peak resident footprint and runs in 110 ms per segment on a server-class CPU, so real-time operation does not require a GPU.

These findings demonstrate that one well-curated training source is sufficient to build a detector that generalizes across disparate hospitals, devices and patient populations with our model architecture, including to ambulatory two-lead recordings it was never trained on. Establishing clinical utility would require prospective validation in the intended care setting, which we identify as the necessary next step rather than a completed one.

### Future Work

We will launch a prospective emergency-department study to quantify clinical impact on alarm burden and time-to-diagnosis. In parallel, we will benchmark the network on resource-constrained edge devices—such as mobile CPUs, ARM-based single-board computers and low-power GPUs—and explore quantization, pruning and knowledge distillation [49] to further reduce inference latency and memory footprint. The ablation reported in Section 3.7 suggests a smaller variant would match the complete architecture on 12-lead input; characterizing the smallest configuration that retains the reduced-lead robustness is a natural next step.

## Figures and Tables

**Table 1:** Datasets used in this study. ^*a*^ Ambulatory two-lead Holter, counted in 10 s windows. ^*b*^ After excluding Ningbo records coded as atrial flutter (Section 3.8); 70,602 before exclusion.

Dataset	Role	AFib	Non-AFib	Total
HDXML	Training	9628	57,804	67,432
*Independent external cohorts*				
MIMIC-IV	External	78,417	721,617	800,034
CODE-15	External	3866	188,202	192,068
CPSC 2021 ^*a*^	External	58,690	114,940	173,630
PhysioNet/CinC 2021 ^*b*^	External	4223	60,306	64,529
SPH	External	502	20,114	20,616
MIT-BIH ^*a*^	External	757	7720	8477
Total external				1,259,354
*Nested within PhysioNet/CinC 2021 (not counted above)*				
Chapman–Shaoxing	Nested	1441	7075	8516
Offline-TS	Nested	294	5306	5600

**Table 2:** Cohorts nested within PhysioNet/CinC 2021, reported separately because their records are contained in that cohort and they are therefore not independent of it. Same model, same protocol, 95% cluster-bootstrap CIs.

Nested Cohort	*n*	AFib	ROC–AUC (95% CI)	Sensitivity (95% CI)	Specificity (95% CI)
Chapman–Shaoxing	8516	1441	0.989 (0.987–0.991)	0.962 (0.951–0.972)	0.966 (0.961–0.970)
Offline-TS (PhysioNet/CinC 2020)	5600	294	0.957 (0.942–0.969)	0.878 (0.840–0.912)	0.918 (0.911–0.925)

**Table 3:** Key elements of the training configuration for the HDXML-only model. The complete configuration, including seed, precision, initialization and hardware, is given in Supplementary Table S2.

Setting	Value
Training/validation split	53,945/13,487 records (80/20, record level); no internal test split, external cohorts only
Optimizer	AdamW, weight decay $1\times 10^{-4}$, gradient clipping 1.0
Learning rate	$1\times 10^{-5}$ embedding network, $1\times 10^{-4}$ remaining modules
Schedule	Cosine annealing, $T_{\max}=20$ epochs, $\eta_{\min}=1\times 10^{-6}$
Budget and selection	200 epochs fixed, no early stopping; minimum validation loss (epoch 57)
Loss	Binary cross-entropy with logits, $w_{+}=2.65$

**Table 4:** Performance of the HDXML-only model on six independently sourced external cohorts: point estimates with 95% confidence intervals from a cluster bootstrap (2000 resamples; resampling unit in Supplementary Table S3). All evaluations used the same checkpoint with no retraining or dataset-specific adjustment. ^*a*^ Ambulatory two-lead Holter, evaluated in 10 s windows; CPSC 2021 gives 173,630 windows from 1435 recordings in 105 patients and MIT-BIH 8477 from 47 subjects, so their intervals resample by patient and are correspondingly wide. ^*b*^ Excludes 6073 Ningbo records coded as atrial flutter (Section 3.8).

Cohort	Source	*n*	AFib	ROC–AUC (95% CI)	Sensitivity (95% CI)	Specificity (95% CI)	PPV
MIMIC-IV	Beth Israel Deaconess, USA (ICU)	800,034	78,417	0.966 (0.965–0.967)	0.753 (0.748–0.759)	0.977 (0.977–0.978)	0.784
CODE-15	Minas Gerais, Brazil	192,068	3866	0.960 (0.956–0.963)	0.583 (0.565–0.600)	0.993 (0.992–0.993)	0.626
CPSC 2021 ^*a*^	Multi-center, China (Holter)	173,630	58,690	0.963 (0.929–0.987)	0.890 (0.819–0.944)	0.903 (0.881–0.923)	0.824
PhysioNet/CinC 2021 ^*b*^	Eight databases, multi-national	64,529	4223	0.978 (0.975–0.981)	0.880 (0.871–0.890)	0.984 (0.983–0.985)	0.796
SPH	Shandong Provincial Hospital, China	20,616	502	0.999 (0.998–0.999)	0.960 (0.942–0.976)	0.996 (0.995–0.997)	0.850
MIT-BIH ^*a*^	Beth Israel, USA (Holter)	8477	757	0.995 (0.988–0.999)	0.992 (0.973–1.000)	0.897 (0.835–0.950)	0.486

**Table 5:** Causal perturbation of the model input, on a class-stratified sample of 20,000 PhysioNet/CinC 2021 records (1308 AFib). The random control occludes the same total duration at random positions.

Occluded Region	ROC–AUC	Sensitivity	ΔROC–AUC	ΔSensitivity
None (reference)	0.9754	0.871	—	—
P-wave interval	0.7074	0.196	0.2680	0.6743
QRS complex	0.7255	0.063	0.2499	0.8081
T wave	0.9563	0.606	0.0191	0.2645
Random intervals, matched duration	0.9617	0.761	0.0137	0.1101

**Table 6:** Comparison with compact 1-D architectures. The six cohort columns are **ROC–AUC** on each external cohort. All four models were trained on HDXML under the identical protocol of Table 3 and evaluated on the same records; each was trained once. Latency is single-threaded CPU inference, on the hardware given in the text.

Model	Params	GMACs	CPU (ms)	MIMIC-IV	CODE-15	CPSC 2021	PhysioNet	SPH	MIT-BIH
This work (14.1 M)	14.07 M	16.66	262	0.9662	0.9598	0.9628	0.9780	0.9989	0.9953
SqueezeNet-1D	0.90 M	0.30	7	0.9649	0.9656	0.9931	0.9799	0.9976	0.9925
MobileNet-1D	1.91 M	0.48	10	0.9623	0.9659	0.9807	0.9794	0.9980	0.9897
ResNet-1D (narrow)	2.90 M	0.63	13	0.9654	0.9658	0.9808	0.9796	0.9983	0.9940

**Table 7:** Probability calibration by cohort at the a priori threshold τ=0.5. ΔF1 is the F1 forgone by retaining 0.5 instead of the cohort’s own optimum $\tau_{F1}$. Full sweep in Supplementary Table S5.

Cohort	Prevalence	Brier	ECE	$\tau_{F1}$	ΔF1
MIMIC-IV	9.8%	0.0385	0.0324	0.350	0.003
CODE-15	2.0%	0.0126	0.0085	0.345	0.006
CPSC 2021	33.8%	0.0767	0.0460	0.375	0.002
PhysioNet 2021	6.5%	0.0192	0.0168	0.655	0.004
SPH	2.4%	0.0042	0.0036	0.735	0.014
MIT-BIH	8.9%	0.0706	0.1075	0.975	0.258

**Table 8:** Component ablation: the four configurations that carry the conclusion, of ten trained. Every arm was trained from scratch on HDXML under the identical protocol of Table 3, so only the architecture varies. ΔROC–AUC is relative to the complete architecture on PhysioNet/CinC 2021 (paired DeLong and Holm-corrected); positive values favor the ablated arm. Latency is single-threaded CPU inference. All ten arms are in Supplementary Table S4.

Configuration	Params (M)	GMACs	CPU (ms)	PhysioNet ROC–AUC (95% CI)	CODE-15	ΔROC–AUC	*p*
Baseline CNN (residual path only)	9.99	12.40	178	0.9412 (0.9379–0.9444)	0.9692	+0.0019	0.136
Complete architecture	14.07	16.66	235	0.9393 (0.9362–0.9425)	0.9519	—	—
− squeeze–excitation (all)	13.40	16.66	234	0.9385 (0.9350–0.9418)	0.9609	−0.0008	0.851
− dense feature stack	12.23	14.36	198	0.9234 (0.9197–0.9267)	0.9402	−0.0159	<0.001

**Table 9:** Contribution of squeeze–excitation as a function of available leads, on PhysioNet/CinC 2021. The two contrasts differ only in the presence of squeeze–excitation, and both models in a pair are scored on the same records, so differences use a paired DeLong test, Holm-corrected, across the five lead configurations. Positive ΔROC–AUC favors squeeze–excitation.

Lead Configuration	Leads	With SE	Without SE	ΔROC–AUC (95% CI)	*p*	Significant
*Complete architecture vs. squeeze–excitation removed throughout*						
12_lead	12	0.9393	0.9385	+0.0008 (−0.0007, +0.0023)	0.568	no
limb_6	6	0.9339	0.9276	+0.0063 (+0.0049, +0.0078)	<0.001	yes
precordial_6	6	0.9356	0.9347	+0.0009 (−0.0008, +0.0027)	0.568	no
2_lead_I_II	2	0.9231	0.9038	+0.0193 (+0.0175, +0.0212)	<0.001	yes
lead_II	1	0.9163	0.8987	+0.0175 (+0.0156, +0.0195)	<0.001	yes
*Baseline CNN with vs. without squeeze–excitation*						
12_lead	12	0.9386	0.9412	−0.0026 (−0.0042, −0.0010)	0.002	yes
limb_6	6	0.9284	0.9184	+0.0101 (+0.0080, +0.0121)	<0.001	yes
precordial_6	6	0.9369	0.9343	+0.0026 (+0.0008, +0.0043)	0.005	yes
2_lead_I_II	2	0.9354	0.9229	+0.0125 (+0.0106, +0.0144)	<0.001	yes
lead_II	1	0.9295	0.9093	+0.0202 (+0.0183, +0.0222)	<0.001	yes

**Table 10:** Variable-lead evaluation on PhysioNet/CinC 2021 (n=64,529; 4223 AFib), using the same checkpoint with no retraining or threshold adjustment. Random configurations are mean ± standard deviation over five seeds; non-inferiority is assessed against a pre-specified margin of 0.02 ROC–AUC. Every configuration is in Supplementary Table S9.

Configuration	Leads	Sensitivity	Specificity	ROC–AUC (95% CI)	ΔROC–AUC	Non-Inferior
12-lead baseline	12	0.880	0.984	0.9780 (0.9750–0.9809)	—	—
9 retained, random	9	0.882±0.008	0.983±0.001	0.9779±0.0006	0.0001	Yes
6 retained, random	6	0.870±0.023	0.982±0.001	0.9773±0.0016	0.0007	Yes
2 retained, random	2	0.805±0.017	0.970±0.007	0.9625±0.0049	0.0156	4/5
Lead II only	1	0.781	0.971	0.9681 (0.9654–0.9707)	0.0099	Yes
Lead I only	1	0.570	0.969	0.9317 (0.9274–0.9356)	0.0463	No

**Table 11:** Performance of recent AFib-detection systems (binary AFib vs. non-AFib unless stated). ‘Ext.’ = external unseen cohort; ‘Int.’ = internal hold-out or CV. Values are those reported by each study; cohorts, label definitions and task scopes differ, so rows are not directly comparable. ‘-’ = information not reported.

Study	Test Type	Dataset(s)	Sens.%	Spec.%	Acc./F1/AUC	Task Scope	Ref.
*Block A—External test-set evaluations*							
This work (HDXML → SPH)	Ext.	SPH	96.02	99.58	ROC-AUC 0.999	AFib vs. Non-AFib	–
This work (HDXML → MIT-BIH)	Ext.	MIT-BIH (2-lead)	99.21	89.72	ROC-AUC 0.995	AFib vs. Non-AFib	–
This work (HDXML → PhysioNet 2021)	Ext.	PhysioNet/CinC 2021	88.04	98.42	ROC-AUC 0.978	AFib vs. Non-AFib	–
This work (HDXML → MIMIC-IV)	Ext.	MIMIC-IV	75.32	97.75	ROC-AUC 0.966	AFib vs. Non-AFib	–
This work (HDXML → CPSC 2021)	Ext.	CPSC 2021 (2-lead)	89.01	90.27	ROC-AUC 0.963	AFib vs. Non-AFib	–
This work (HDXML → CODE-15)	Ext.	CODE-15	58.28	99.28	ROC-AUC 0.960	AFib vs. Non-AFib	–
Knowledge-embedded pseudo-Siamese CNN	Ext.	Chapman	–	–	99.75 Acc.	AFib/AFL/N	[40]
Robust DL across geography	Ext.	CPSC 2021 (+geo sets)	–	–	F1 ≈ 0.90–0.95	AFib ± AFL vs. N	[41]
CLA-AF	Ext.	CPSC 2018; PhysioNet 2021	–	–	ROC-AUC > 0.95	AFib/N/O	[42]
ECG-AI for new-onset AFib/AFL	Ext.	MIMIC-IV	–	–	ROC-AUC 0.77	Risk pred.	[39]
Explainable DLM	Ext.	Chapman	–	–	ROC-AUC 0.997–0.999	AFib only	[43]
Modified CNN (paroxysmal AFib)	Ext.	CODE (15%)	–	–	ROC-AUC 0.884	Paroxysmal	[44]
*Block B – Internal (train/validation) evaluations*							
This work (multi-dataset CV)	Int.	7 combined corpora	90.08	97.93	97.54 Acc.	AFib vs. Non-AFib	–
Rajpurkar et al. 2017	Int.	Stanford 1-lead	–	–	F1 78.7	12 rhythms	[17]
Attia et al. 2019	Int.	Mayo Clinic (649k)	79.0	79.0	ROC-AUC 0.87	Latent AFib	[19]
Ribeiro et al. 2020	Int.	Tele-health (2.3M)	–	99.0	F1 > 80	6 rhythms	[20]
Cai et al. 2020	Int.	16,557 mixed sources	99.19	99.44	99.35 Acc.	AFib only	[45]
Quantifying DNN uncertainty	Int.	Chapman	–	–	F1 0.64–0.67	AFib vs. Non-AFib	[46]
End-to-end AFib risk (CODE)	Int.	CODE	–	–	ROC-AUC 0.845	Risk pred.	[47]
MI-capsule CNN	Int.	Chapman	–	–	97.4 Acc.	Multi-arrhythmia	[48]

## Data Availability

All datasets used in this study are publicly available third-party datasets, cited in the references: HDXML [22], CODE-15 [24], CPSC 2021 [25], PhysioNet/CinC 2021 [26], the SPH database [27], the MIT-BIH Arrhythmia Database [28], Chapman–Shaoxing [29], the PhysioNet/CinC 2020 challenge set [30], MedalCare-XL [31] and MIMIC-IV-ECG [23]. The expanded PhysioNet release of the Chapman–Shaoxing and Ningbo collections [50] is the source of the Chapman–Shaoxing records contained within PhysioNet/CinC 2021. No new data were created for this study.

## Supplementary Materials

The following supporting information can be downloaded at https://www.mdpi.com/article/10.3390/bioengineering13101174/s1, Section S1: The three secondary experiments (multi-dataset, P-wave-mask and vectorcardiogram training), with Table S1 consolidating all their reported metrics and Figures S1 and S2. Table S2: Complete training configuration. Table S3: Bootstrap resampling unit and cluster count by cohort. Table S4: Complete component ablation, all ten configurations. Table S5: Complete calibration and threshold sweep. Table S6: Sensitivity of PhysioNet/CinC 2021 performance to the treatment of Ningbo flutter-coded records. Tables S7 and S8: Confusion matrices and prevalence-standardized predictive values for every external cohort. Table S9 and Figures S3 and S4: Complete variable-lead evaluation, including all fixed anatomical subsets and individual leads.

## Abbreviations

The following abbreviations are used in this manuscript:

AFib	Atrial fibrillation
ECG	Electrocardiogram
CNN	Convolutional neural network
SE	Squeeze-and-excitation
VCG	Vectorcardiogram
ICU	Intensive care unit
AUROC/ROC–AUC	Area under the receiver operating characteristic curve
PPV/NPV	Positive/negative predictive value
ECE	Expected calibration error
IG	Integrated Gradients
MAC/GMAC	Multiply–accumulate operation/$10^{9}$ of them
FLOP	Floating-point operation (1 MAC = 2 FLOPs)
FP32	32-bit single-precision floating-point arithmetic
CPU/GPU	Central/graphics processing unit
MLII	Modified limb lead II (Holter recordings)
HDXML	Heart Disease XML Strain dataset
MIMIC-IV	Medical Information Mart for Intensive Care, version IV
