# Supplementary material for: A Fast and Generalizable Deep Neural Network for the Detection of Atrial Fibrillation
Source: Bioengineering (Basel). 2026 Oct 8;13(10):1174. doi: 10.3390/bioengineering13101174 (PMC13701297; doi:10.3390/bioengineering13101174)
Supplement: Supplementary file 1 [file bioengineering-13-01174-s001.zip › bioengineering-4585711-supplementary.pdf]

Supplementary Materials

A Fast and Generalizable Deep Neural Network for the Detection of Atrial Fibrillation

This document contains supporting material for the main article: the secondary experiments conducted en route to the final model, the complete versions of tables that appear in condensed form in the main text, and the full per-cohort grids. Section, table and figure numbers prefixed with **S** refer to this document; unprefixed numbers refer to the main article.

Contents

S1 Secondary Experiments 1

S1.1 Multi-Dataset Training . . . 2

S1.1.1 Performance on the Held-Out Internal Split . . . 2

S1.1.2 Evaluation on MIMIC-IV . . . 2

S1.2 Why These Experiments Were Not Carried Forward . . . 3

S1.2.1 P-wave Mask Augmented Classifier . . . 3

S1.2.2 Vectorcardiogram (VCG)-Based AFib Detection . . . 3

S2 Complete Training Configuration 3

S3 Statistical Methods 4

S4 Complete Component Ablation 5

S5 Complete Calibration and Threshold Sensitivity 5

S6 Label Coding in the Ningbo Subset of PhysioNet/CinC 2021 6

S7 Error Structure and Predictive Values 6

S8 Complete Variable-Lead Evaluation 7

S1 Secondary Experiments

Three experimental protocols preceded the single-source model reported in the main article. They are recorded here because they motivated the final design: the multi-dataset experiment (Experiment 1) established the task and the evaluation pipeline, and the P-wave-mask (Experiment 2) and vectorcardiogram (Experiment 3) variants tested two hypotheses about how to inject domain knowledge, neither of which improved on the simpler model. The main article's result — that training on the single curated HDXML corpus generalizes across six external cohorts — is what followed from these.

All three use the architecture, preprocessing and augmentation of Sections 2.1–2.3 of the main article, and all were evaluated at $\tau = 0.7$, chosen to favor specificity in a continuous-monitoring context. This differs from the $\tau = 0.5$ used for every result in the main article, so the figures below are *not* directly comparable with those in Table 4 of the main article; Table S1 therefore includes the HDXML-only model on MIMIC-IV as a reference row.

Table S1 consolidates every metric reported for these three experiments.

Table S1: All metrics reported for the three secondary experiments, together with the HDXML-only model on MIMIC-IV for reference. Experiments 1–3 were evaluated at $\tau = 0.7$; the reference row is at $\tau = 0.5$. Dashes mark quantities the original analysis did not report. Experiment 3 was quantified only as a change relative to the same architecture without the vectorcardiogram transform, so it has no absolute entry here and is described in Section S1.2.

**Experiment**	**Evaluation set**	**Acc.**	**Sens.**	**Spec.**	**Prec.**	**F1**	τ
1. Multi-dataset	Held-out internal split (n = 29,740)	97.54	90.08	97.93	84.36	88.50	0.7
1. Multi-dataset	MIMIC-IV (n = 800,034)	95.71	70.35	98.46	83.26	76.26	0.7
2. P-wave mask	MIMIC-IV (n = 800,034)	95.36	67.80	98.35	—	—	0.7
HDXML-only (main article)	MIMIC-IV (n = 800,034)	—	75.32	97.75	78.41	76.84	0.5

The multi-dataset model was trained on the union of CODE-15, Chapman-Shaoxing, SPH, MedalCare-XL, the PhysioNet/CinC 2020 challenge set and PhysioNet/CinC 2021, split 237,921 / 29,740 / 29,740 into training, validation and test at the record level.

S1.1 Multi-Dataset Training

S1.1.1 Performance on the Held-Out Internal Split

We evaluated our model using standard classification metrics including precision, recall, F1-score, and area under the receiver operating characteristic curve (AUROC). The PR curve and AUC-ROC curve can be seen in (Figure S1a, S1b) respectively, which illustrates the classification performance across positive and negative cases.

The model achieved an overall accuracy of 97.54% on the test set, with a precision of 84.36% and recall of 90.08% for atrial fibrillation detection. The F1-score, which represents the harmonic mean of precision and recall, was 88.50%. Sensitivity and specificity were reported to be 90.08% and 97.93% respectively. The model was evaluated with 0.7 threshold of sigmoid activation function.

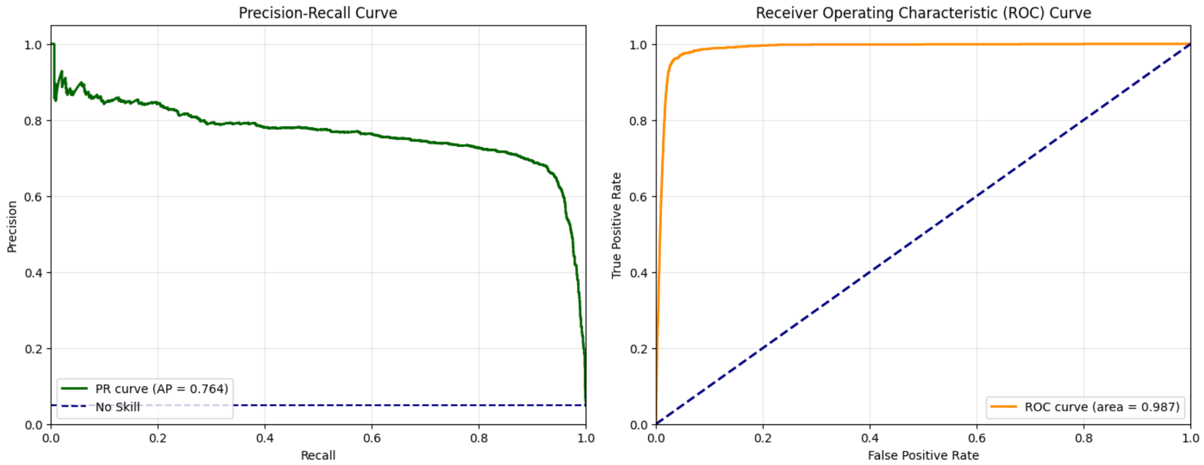

(a) (b)

Figure S1: Performance evaluation metrics for multi-dataset training: (**a**) Precision-Recall curve demonstrating model performance across different thresholds. (**b**) ROC curve illustrating the trade-off between sensitivity and specificity.

These figures are consolidated in Table S1.

S1.1.2 Evaluation on MIMIC-IV

Evaluated on MIMIC-IV, the *multi-dataset* model of Experiment 1 achieved an overall accuracy of 95.71%, with a sensitivity of 70.35% and a specificity of 98.46%. These values describe the multi-dataset model only; the HDXML-only model reported in Table 4 of the main article achieves 75.3% sensitivity and 97.7%

specificity on the same cohort, and the two should not be conflated. The model's precision for AFib detection was 83.26%. The F1-score was computed as 76.26%.

This evaluation ran on a GPU in batches; the 765.63 s total is throughput over all 800,034 records, not per-segment latency. Per-segment CPU latency is reported in Section 4.1 of the main article.

Precision–recall and ROC curves for this evaluation are shown in Figures S2a and S2b.

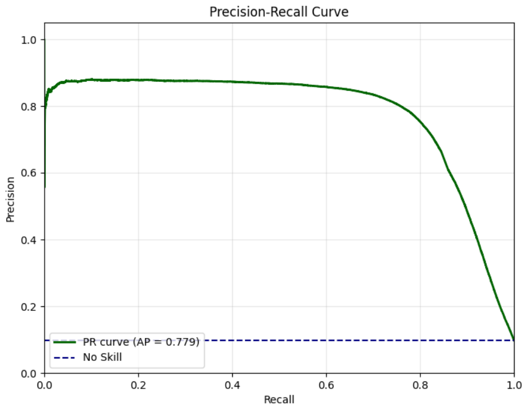

(a)

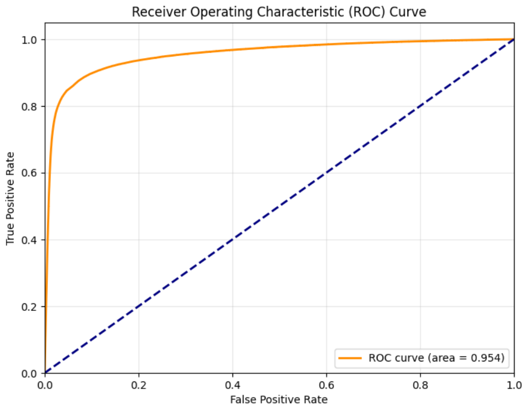

(b)

Figure S2: MIMIC-IV external validation results: (**a**) Precision-Recall curve demonstrating model robustness across thresholds. (**b**) ROC curve illustrating discriminative performance on external dataset.

S1.2 Why These Experiments Were Not Carried Forward

Several supplementary experiments were conducted en route to finalizing the HDXML-only model, which helped justify key architectural and design decisions.

S1.2.1 P-wave Mask Augmented Classifier

A two-stage pipeline segmented P-wave regions with a compact 1-D U-Net and an attention module directed the classifier towards those masks. On MIMIC-IV this reached 95.36% accuracy with 67.80% sensitivity and 98.35% specificity — lower on every metric than the multi-dataset model on the same cohort (95.71%, 70.35%, 98.46%) and than the HDXML-only model that was ultimately adopted. We concluded that the architecture learns to attend to the P-wave region without handcrafted segmentation, which the occlusion analysis in Section 3.4 of the main article later confirmed directly.

S1.2.2 Vectorcardiogram (VCG)-Based AFib Detection

This approach improved robustness on certain datasets, particularly the Offline-TS set, where accuracy increased by 3.89% and F1-score by 12.49%. However, this came at the cost of decreased sensitivity across all datasets. Thus, while promising for noise resilience, VCG-based models require further refinement to match the sensitivity of multi-lead ECG models.

S2 Complete Training Configuration

Table S2 gives the full configuration of the HDXML-only model. The main article reproduces the six load-bearing rows in its Section 2.3.4; everything else is recorded here, including the compute environment, so that the run can be reproduced exactly.

Two points that the configuration table alone does not convey. First, model selection was a genuine minimum rather than a tie: validation loss reached 0.23498 at epoch 57 against 0.23517 at epoch 92 and 0.23592 at epoch 61, so the released checkpoint is the lowest of the 200-epoch run and not merely the earliest of several equal values. Second, the training cost depends almost entirely on the input pipeline: decoding the

HDXML corpus from source on every epoch takes roughly 28 minutes per epoch, whereas the pre-decoded fp16 memmap cache used here reduces this to 1–2 minutes, and it is that cache rather than the accelerator that makes the ablation and baseline runs affordable.

Table S2: Complete training configuration and compute environment for the HDXML-only model. The upper block extends the six rows given in Table 3 of the main article; the lower block records the environment, which the main article does not describe. All environment values were read from the training host rather than transcribed from notes.

Setting	Value
Random seed	42
Training / validation split	53,945 / 13,487 records (80/20, record level)
Held-out test split	none (external cohorts only)
Batch size	32
Optimizer	AdamW
Weight decay	1×10^{-4}
Learning rate, embedding network	1×10^{-5}
Learning rate, remaining modules	1×10^{-4}
Learning rate schedule	Cosine annealing, $T_{\max} = 20$ epochs, $\eta_{\min} = 1 \times 10^{-6}$, stepped per epoch
Gradient clipping	1.0
Numerical precision	16-bit mixed
Weight initialization	Random (framework default)
Training budget	200 epochs, fixed (no early stopping applied)
Model selection criterion	Minimum validation loss (epoch 57)
Loss function	Binary cross-entropy with logits, $w_+ = 2.65$
Deterministic kernels	Not enforced
Best validation loss	0.23498 (epoch 57 of 200)
Compute environment	
Accelerator	1 × NVIDIA H100 80GB HBM3 (single-GPU run; host carries 8)
GPU driver / CUDA / cuDNN	550.90.07 / 12.4 / 9.1.0
Host CPU	2 × Intel Xeon Platinum 8481C @ 2.70 GHz (104 physical cores, 208 threads, AVX-512)
Host memory	1.8 TB
Operating system	Debian GNU/Linux 11 (bullseye)
Framework	PyTorch 2.5.1+cu124, PyTorch Lightning 2.4.0
Input pipeline	Pre-decoded fp16 memmap cache, 7.6 GiB; 1–2 min per epoch against ≈28 min decoding from source

S3 Statistical Methods

Confidence intervals. All intervals are 95% percentile intervals from a cluster (hierarchical) bootstrap with 2000 resamples. The resampling unit is the patient wherever a patient identifier exists, and otherwise the recording or subject. Treating every prediction as independent would materially understate uncertainty on the ambulatory cohorts, where 173,630 and 8477 analysis windows derive from only 105 patients and 47 subjects respectively. Table S3 lists the unit and the resulting cluster count for every cohort.

Paired comparisons. Where two models are scored on identical records — the component ablation of Section S4 and the squeeze-excitation contrasts of the main article — differences in ROC–AUC are tested with the DeLong test for correlated ROC curves, and p-values are Holm-corrected across the family of comparisons. No significance test is reported *between* cohorts: those are different populations with different prevalences and acquisition protocols, so a difference between them is not a statement about the model.

Table S3: Bootstrap resampling unit by cohort.

Cohort	Resampling unit	Units
MIMIC-IV	Patient	161,352
CODE-15	Patient	151,595
SPH	Patient	20,616
PhysioNet/CinC 2021	Record	64,529
CPSC 2021	Patient	105
MIT-BIH	Subject	47

S4 Complete Component Ablation

Table S4 reports all ten configurations. The main article summarizes the four that carry the conclusion — the baseline convolutional path, the complete architecture, and the removal of the dense feature stack and of squeeze-excitation — and the remaining six are given here.

Two observations that the condensed table cannot show. First, the five add-one-component arms and the five remove-one-component arms agree with each other: no component appears beneficial when added and harmful when removed, or vice versa, which is the internal consistency check on the ablation. Second, several arms reach nominal significance with effect sizes of order 0.003 ROC–AUC. These are significant only because the evaluation cohort contains 70,602 records, and are reported with Holm-corrected p-values so that the distinction between statistical and practical significance stays visible.

Table S4: Component ablation. All ten configurations were trained from scratch on HDXML under the identical protocol of Table S2 — same seed, split, optimizer, schedule, augmentation and 80-epoch budget — so only the architecture varies. The upper block adds one component at a time to a baseline of the convolutional (residual) path alone; the lower block removes one component from the complete architecture. ΔROC–AUC is relative to the complete architecture on PhysioNet/CinC 2021, with a paired DeLong test and Holm correction across the nine comparisons; positive values favor the ablated arm. Evaluation uses the full cohort (n = 70,602) without the exclusion of Section S6, which affects every arm equally. Latency is single-threaded CPU inference.

Configuration	Params (M)	GMACs	CPU (ms)	PhysioNet ROC–AUC (95% CI)	CODE-15	ΔROC–AUC	p
Baseline CNN (residual path only)	9.99	12.40	178	0.9412 (0.9379–0.9444)	0.9692	+0.0019	0.136
+ dense path	10.25	12.72	187	0.9378 (0.9345–0.9412)	0.9594	-0.0015	0.258
+ squeeze-excitation	10.05	12.40	172	0.9386 (0.9350–0.9420)	0.9656	-0.0007	0.851
+ multi-scale fusion	13.73	16.33	217	0.9422 (0.9391–0.9451)	0.9734	+0.0029	0.001
**Complete architecture**	14.07	16.66	235	0.9393 (0.9362–0.9425)	0.9519	—	—
− dense path	13.74	16.33	229	0.9418 (0.9384–0.9450)	0.9596	+0.0025	0.053
− squeeze-excitation (all)	13.40	16.66	234	0.9385 (0.9350–0.9418)	0.9609	-0.0008	0.851
− multi-scale fusion	10.33	12.72	181	0.9420 (0.9392–0.9451)	0.9683	+0.0027	0.001
− global-context SE	14.06	16.66	216	0.9388 (0.9353–0.9420)	0.9545	-0.0005	0.851
− dense feature stack	12.23	14.36	198	0.9234 (0.9197–0.9267)	0.9402	-0.0159	<0.001

S5 Complete Calibration and Threshold Sensitivity

Table S5 gives the full threshold sweep. The main article reports expected calibration error, Brier score and the F1 forgone at $\tau = 0.5$; this table adds sensitivity and specificity at $\tau \in \{0.3, 0.5, 0.7\}$ for every cohort, together with the F1-maximizing and Youden-optimal thresholds.

The threshold $\tau = 0.5$ was fixed a priori and no selection procedure was ever applied to it. The F1-optimal threshold ranges from 0.345 on CODE-15 to 0.975 on MIT-BIH, tracking each cohort's prevalence (2.0%–33.8%) as much as the model's behavior, so no single value is optimal across cohorts and selecting one on the evaluation data would have compromised the independence of the external validation.

Table S5: Probability calibration and the sensitivity of reported metrics to the classification threshold. The threshold $\tau = 0.5$ was fixed a priori and never tuned; the columns to its right show how sensitivity and specificity move with τ. The final two columns give the F1-maximizing threshold per cohort and the F1 forgone by using 0.5 instead. That optimum depends on prevalence, which ranges from 2.0% to 33.8% here, so no single value is optimal across cohorts.

				$\tau = 0.3$		$\tau = 0.5$		$\tau = 0.7$		
**Cohort**	**Prev.**	**Brier**	**ECE**	**Sens**	**Spec**	**Sens**	**Spec**	**Sens**	τ_{F1}	**ΔF1**
MIMIC-IV	9.8%	0.0385	0.0324	0.780	0.974	0.753	0.977	0.723	0.350	0.003
CODE-15	2.0%	0.0126	0.0085	0.638	0.990	0.583	0.993	0.518	0.345	0.006
CPSC 2021	33.8%	0.0767	0.0460	0.931	0.877	0.890	0.903	0.840	0.375	0.002
PhysioNet 2021	6.5%	0.0192	0.0168	0.899	0.981	0.880	0.984	0.859	0.655	0.004
SPH	2.4%	0.0042	0.0036	0.966	0.995	0.960	0.996	0.948	0.735	0.014
MIT-BIH	8.9%	0.0706	0.1075	0.993	0.864	0.992	0.897	0.988	0.975	0.258

S6 Label Coding in the Ningbo Subset of PhysioNet/CinC 2021

During analysis we identified a labeling inconsistency in the Ningbo database, which contributes 27,865 of the 70,602 PhysioNet/CinC 2021 records. Across all 34,905 Ningbo records the release assigns atrial flutter (SNOMED 164890007) to 7615 records and atrial fibrillation (164889003) to none. A clinical corpus of this size containing no atrial fibrillation is implausible, since fibrillation is substantially more common than flutter.

The model's output distribution supports this reading. On the Ningbo records coded as flutter, predicted probabilities had a median of 0.992 and 79.9% exceeded 0.5, closely matching its behavior on confirmed AFib elsewhere (median 0.996 on Chapman-Shaoxing) and unlike its behavior on flutter in other databases within the same release (median 0.199 on Chapman-Shaoxing, 0.022 on Georgia, 0.004 on PTB-XL). On the remainder of Ningbo the model behaved normally, with 1.3% of records exceeding 0.5. These records accounted for 5130 of the 5803 false positives (88.4%) observed on PhysioNet/CinC 2021 under the original labels.

We therefore exclude the 6073 flutter-coded Ningbo records present in our evaluation split, which is the conservative option: it removes ambiguous labels rather than reassigning them. Table S6 reports the alternatives. Discrimination is essentially unchanged across all four policies, so the conclusion does not depend on which is adopted.

Table S6: Sensitivity of PhysioNet/CinC 2021 performance to the treatment of Ningbo flutter-coded records.

**Label policy**	n	**Sensitivity**	**Specificity**	**ROC–AUC**
As released	70,602	0.880	0.913	0.939
Ningbo flutter-coded excluded (adopted)	64,529	0.880	0.984	0.978
Ningbo flutter-coded counted as AFib	70,602	0.832	0.984	0.977
All flutter-coded records excluded	63,902	0.883	0.988	0.980

S7 Error Structure and Predictive Values

Table S7 reports the full confusion matrix for each external cohort. Because positive predictive value depends on prevalence, which ranges from 2.0% in CODE-15 to 33.8% in CPSC 2021, the observed PPVs are not directly comparable between cohorts; Table S8 therefore standardizes predictive values to fixed reference prevalences.

Two patterns are worth noting. First, errors are asymmetric and cohort-dependent: on CODE-15 the model is highly specific (99.3%) but detects only 58.3% of AFib cases, whereas on MIT-BIH it detects 99.2% at the cost of a lower specificity of 89.7%. Second, at the low prevalences typical of community screening, specificity rather than sensitivity dominates achievable PPV. At $\pi = 2\%$ the CODE-15 operating point yields a PPV of 0.63 while the PhysioNet/CinC 2021 operating point yields 0.53, despite the latter having far higher sensitivity. Any screening deployment would need to select its operating point against the prevalence of the intended population rather than against discrimination alone.

Table S7: Confusion matrices and predictive values at $\tau = 0.5$ for each external cohort. Prevalence is the observed AFib rate in that cohort. PPV and NPV are prevalence-dependent and are therefore not comparable across cohorts; Table S8 standardizes them.

Cohort	TN	FP	FN	TP	Prevalence	PPV	NPV
MIMIC-IV	705,357	16,260	19,353	59,064	9.8%	0.784	0.973
CODE-15	186,855	1347	1613	2253	2.0%	0.626	0.991
CPSC 2021	103,759	11,181	6448	52,242	33.8%	0.824	0.941
PhysioNet/CinC 2021	59,352	954	505	3718	6.5%	0.796	0.992
SPH	20,029	85	20	482	2.4%	0.850	0.999
MIT-BIH	6926	794	6	751	8.9%	0.486	0.999

Table S8: Predictive values (PPV / NPV) standardized to reference AFib prevalences using each cohort's measured sensitivity and specificity. The 1–2% range corresponds to community screening and 5–10% to inpatient or critical-care populations.

Cohort	$\pi = 1\%$	$\pi = 2\%$	$\pi = 5\%$	$\pi = 10\%$
MIMIC-IV	0.252 / 0.997	0.406 / 0.995	0.638 / 0.987	0.788 / 0.973
CODE-15	0.451 / 0.996	0.624 / 0.991	0.811 / 0.978	0.900 / 0.955
CPSC 2021	0.085 / 0.999	0.157 / 0.998	0.325 / 0.994	0.504 / 0.987
PhysioNet/CinC 2021	0.360 / 0.999	0.532 / 0.998	0.745 / 0.994	0.861 / 0.987
SPH	0.697 / 1.000	0.823 / 0.999	0.923 / 0.998	0.962 / 0.996
MIT-BIH	0.089 / 1.000	0.164 / 1.000	0.337 / 1.000	0.517 / 0.999

S8 Complete Variable-Lead Evaluation

Table S9 gives every lead configuration evaluated on PhysioNet/CinC 2021 ($n = 64{,}529$), and Figures S3 and S4 present the fixed anatomical subsets and the full sensitivity/specificity/ROC–AUC grid respectively. The main article reports the 12-, 9-, 6- and 2-lead conditions and the strongest single lead.

Every configuration uses the same checkpoint with no retraining and no threshold adjustment: masked leads are set to zero at their standard 12-lead positions. This is the same intervention used for the occlusion analysis of Section 3.4 of the main article, so the two are directly comparable.

Table S9: Variable-lead evaluation on PhysioNet/CinC 2021 ($n = 64{,}529$; 4223 AFib). Every configuration is scored on the same records with the same checkpoint; missing leads are zero-filled at inference and no parameter or threshold was changed. ΔROC–AUC is relative to the 12-lead baseline. Non-inferiority is a one-sided paired DeLong test against a pre-specified margin of 0.02 ROC–AUC; two-sided tests are Holm-corrected across configurations. Random-masking rows are mean ± SD over five seeds.

Configuration	Leads	Sensitivity	Specificity	ROC–AUC (95% CI)	ΔROC–AUC	Non-inferior
12-lead baseline	12	0.880	0.984	0.9780 (0.9750–0.9809)	—	—
9 retained, random	9	0.882 ± 0.008	0.983 ± 0.001	0.9779 ± 0.0006	0.0001	**Yes**
6 retained, random	6	0.870 ± 0.023	0.982 ± 0.001	0.9773 ± 0.0016	0.0007	**Yes**
2 retained, random	2	0.805 ± 0.017	0.970 ± 0.007	0.9625 ± 0.0049	0.0156	4/5
Limb leads I–aVF	6	0.858	0.970	0.9721 (0.9691–0.9749)	0.0060	**Yes**
Precordial V1–V6	6	0.880	0.977	0.9755 (0.9725–0.9784)	0.0025	**Yes**
Lead II only	1	0.781	0.971	0.9681 (0.9654–0.9707)	0.0099	**Yes**
Lead I only	1	0.570	0.969	0.9317 (0.9274–0.9356)	0.0463	No
Lead aVF only	1	0.611	0.938	0.9112 (0.9068–0.9152)	0.0668	No
Lead III only	1	0.330	0.909	0.7786 (0.7717–0.7855)	0.1994	No

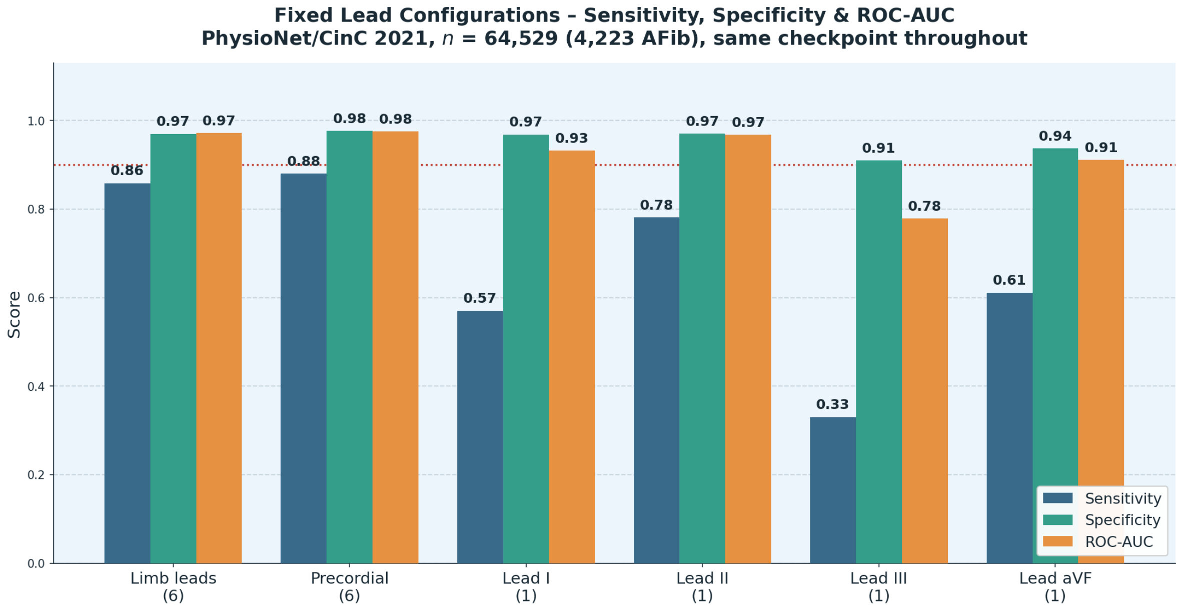

Figure S3: Performance across fixed anatomical lead subsets and individual leads on PhysioNet/CinC 2021 ($n =$ 64,529). The dashed horizontal line marks the 0.90 reference. Both six-lead subsets and Lead II achieve discrimination non-inferior to full 12-lead input; Leads I, aVF and III do not.

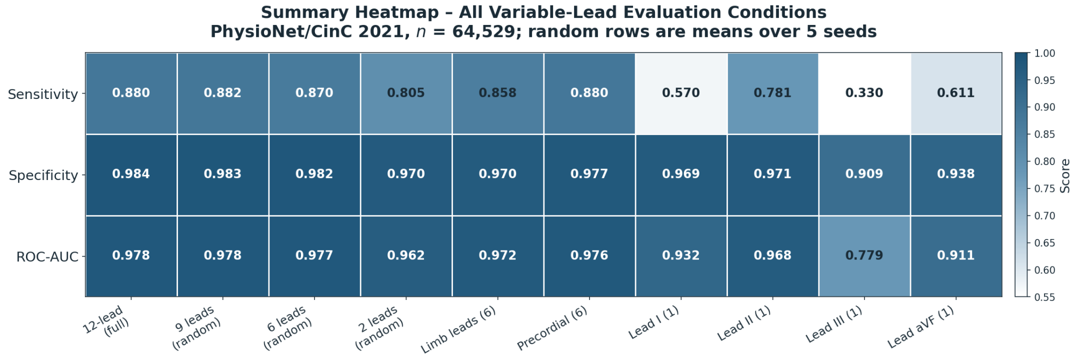

Figure S4: Heatmap of sensitivity, specificity and ROC–AUC across all variable-lead conditions on PhysioNet/CinC 2021. Darker blue indicates higher scores (scale 0.55–1.00). Specificity is markedly more stable than sensitivity under masking, the pattern expected when a detector loses signal rather than gains noise.
